# Tuberculosis-associated IFN-I induces Siglec-1 on tunneling nanotubes and favors HIV-1 spread in macrophages

**DOI:** 10.7554/eLife.52535

**Published:** 2020-03-30

**Authors:** Maeva Dupont, Shanti Souriant, Luciana Balboa, Thien-Phong Vu Manh, Karine Pingris, Stella Rousset, Céline Cougoule, Yoann Rombouts, Renaud Poincloux, Myriam Ben Neji, Carolina Allers, Deepak Kaushal, Marcelo J Kuroda, Susana Benet, Javier Martinez-Picado, Nuria Izquierdo-Useros, Maria del Carmen Sasiain, Isabelle Maridonneau-Parini, Olivier Neyrolles, Christel Vérollet, Geanncarlo Lugo-Villarino

**Affiliations:** 1 Institut de Pharmacologie et Biologie Structurale, IPBS, Université de Toulouse, CNRS, UPSToulouseFrance; 2 International associated laboratory (LIA) CNRS 'IM-TB/HIV'ToulouseFrance; 3 Institute of Experimental Medicine-CONICET, National Academy of MedicineBuenos AiresArgentina; 4 Aix Marseille Univ, CNRS, INSERM, CIMLMarseilleFrance; 5 Tulane National Primate Research Center, Department of Microbiology and Immunology, School of Medicine, Tulane UniversityCovingtonUnited States; 6 IrsiCaixa AIDS Research Institute, Department of RetrovirologyBadalonaSpain; 7 Universitat Autònoma de BarcelonaBarcelonaSpain; 8 University of Vic-Central University of Catalonia (UVic-UCC)VicSpain; 9 Catalan Institution for Research and Advanced Studies (ICREA)BarcelonaSpain; 10 Institut d’Investigació en Ciències de la Salut Germans Trias i PujolBadalonaSpain; University of the WitwatersrandSouth Africa; University of HelsinkiFinland

**Keywords:** macrophages, HIV, mycobacterium tuberculosis, tunneling natotubes, interferons, co-infection, Human, Rhesus macaque

## Abstract

While tuberculosis (TB) is a risk factor in HIV-1-infected individuals, the mechanisms by which *Mycobacterium tuberculosis* (Mtb) worsens HIV-1 pathogenesis remain scarce. We showed that HIV-1 infection is exacerbated in macrophages exposed to TB-associated microenvironments due to tunneling nanotube (TNT) formation. To identify molecular factors associated with TNT function, we performed a transcriptomic analysis in these macrophages, and revealed the up-regulation of Siglec-1 receptor. Siglec-1 expression depends on Mtb-induced production of type I interferon (IFN-I). In co-infected non-human primates, Siglec-1 is highly expressed by alveolar macrophages, whose abundance correlates with pathology and activation of IFN-I/STAT1 pathway. Siglec-1 localizes mainly on microtubule-containing TNT that are long and carry HIV-1 cargo. Siglec-1 depletion decreases TNT length, diminishes HIV-1 capture and cell-to-cell transfer, and abrogates the exacerbation of HIV-1 infection induced by Mtb. Altogether, we uncover a deleterious role for Siglec-1 in TB-HIV-1 co-infection and open new avenues to understand TNT biology.

## Introduction

Co-infection with *Mycobacterium tuberculosis* (Mtb) and the human immunodeficiency virus (HIV-1), the agents of tuberculosis (TB) and acquired immunodeficiency syndrome (AIDS), respectively, is a major health issue. Indeed, TB is the most common illness in HIV-1-infected individuals, about 55% of TB notified patients are also infected with HIV-1, and about a fifth of the TB death toll occurs in HIV-1 co-infected individuals (WHO health report 2017). Clinical studies evidence a synergy between these two pathogens, which is associated with a spectrum of aberration in immune function (Esmail et al., 2018). Yet, while progress has been made towards understanding how HIV-1 enhances Mtb growth and spread, the mechanisms by which Mtb exacerbates HIV-1 infection require further investigation (Bell and Noursadeghi, 2018; Deffur et al., 2013; Diedrich and Flynn, 2011).

Besides CD4^+^ T cells, macrophages are infected by HIV-1 in humans and by the simian immunodeficiency virus (SIV), the most closely related lentivirus to HIV, in non-human primates (NHP) (Cribbs et al., 2015; Rodrigues et al., 2017). Recently, using a humanized mouse model, macrophages were shown to sustain HIV-1 infection and replication, even in the absence of T cells (Honeycutt et al., 2017; Honeycutt et al., 2016). This is in line with several studies characterizing tissue macrophages, such as alveolar, microglia and gut macrophages, as reservoirs in HIV-1 patients undergoing antiretroviral therapy (Ganor et al., 2019; Jambo et al., 2014; Mathews et al., 2019; Sattentau and Stevenson, 2016).

Macrophages are key host cells for Mtb (O'Garra et al., 2013; VanderVen et al., 2016). We recently reported the importance of macrophages in HIV-1 exacerbation within the TB co-infection context (Souriant et al., 2019). Using relevant in vitro and in vivo models, we showed that TB-associated microenvironments activate macrophages towards an M(IL-10) profile, distinguished by a CD16^+^CD163^+^MerTK^+^ phenotype. Acquisition of this phenotype is dependent on the IL-10/STAT3 signaling pathway (Lastrucci et al., 2015). M(IL-10) macrophages are highly susceptible not only to Mtb infection (Lastrucci et al., 2015), but also to HIV-1 infection and spread (Souriant et al., 2019). At the functional level, we demonstrated that TB-associated microenvironments stimulate the formation of tunneling nanotubes (TNT), membranous channels connecting two or more cells over short to long distances above substrate. TNT are subdivided in two classes based on their thickness and cytoskeleton composition: ‘thin’ TNT (<0.7 μm in diameter) containing F-actin, and ‘thick’ TNT (>0.7 μm in diameter) are enriched in F-actin and microtubules (MT) (Souriant et al., 2019). Thick TNT are functionally distinguished by the transfer of large organelles, such as lysosomes and mitochondria (Dupont et al., 2018; Eugenin et al., 2009; Hashimoto et al., 2016). While the contribution for each TNT class to HIV-1 pathogenesis has not been explored (Dupont et al., 2018; Eugenin et al., 2009; Hashimoto et al., 2016), we reported that total inhibition of TNT formation in M(IL-10) macrophages resulted in the abrogation of HIV-1 exacerbation induced by Mtb (Souriant et al., 2019). Factors influencing TNT function in M(IL-10) macrophages remain unknown at large.

In this study, global mapping of the M(IL-10) macrophage transcriptome revealed Siglec-1 (CD169, or sialoadhesin) as a potential factor responsible for HIV-1 dissemination in the co-infection context with TB. As a type-I transmembrane lectin receptor, Siglec-1 possesses a large extracellular domain composed of 17 immunoglobulin-like domains, including the N-terminal V-set domain, which allows the *trans* recognition of terminal α2,3-linked sialic acid residues in *O*- and *N*-linked glycans and glycolipids, such as those surface-exposed in HIV-1 and SIV particles (Izquierdo-Useros et al., 2012a; Puryear et al., 2012). While Siglec-1 has yet to be implicated in the TB context, it is clearly involved in the pathogenesis of HIV-1, SIV and other retroviruses (Martinez-Picado et al., 2017). Siglec-1 is mainly expressed in myeloid cells (*e.g.* macrophages and dendritic cells) and participates in HIV-1 transfer from myeloid cells to T cells, as well as in the initiation of virus-containing compartment (VCC) formation in macrophages (Izquierdo-Useros et al., 2012a; Izquierdo-Useros et al., 2012b; Puryear et al., 2013; Puryear et al., 2012), and in the viral dissemination in vivo (Akiyama et al., 2017; Izquierdo-Useros et al., 2012a; Sewald et al., 2015). Indeed, HIV-1 and other retroviruses have evolved the capacity to hijack the immune surveillance and housekeeping immunoregulatory functions of Siglec-1 (Izquierdo-Useros et al., 2014; O'Neill et al., 2013). Here, we investigate how Siglec-1 expression is induced by TB, and the role it has in the capture and transfer of HIV-1 by TB-induced M(IL-10) macrophages, in particular in the context of TNT.

## Results

### Tuberculosis-associated microenvironments induce Siglec-1 in macrophages

**Figure 1.**
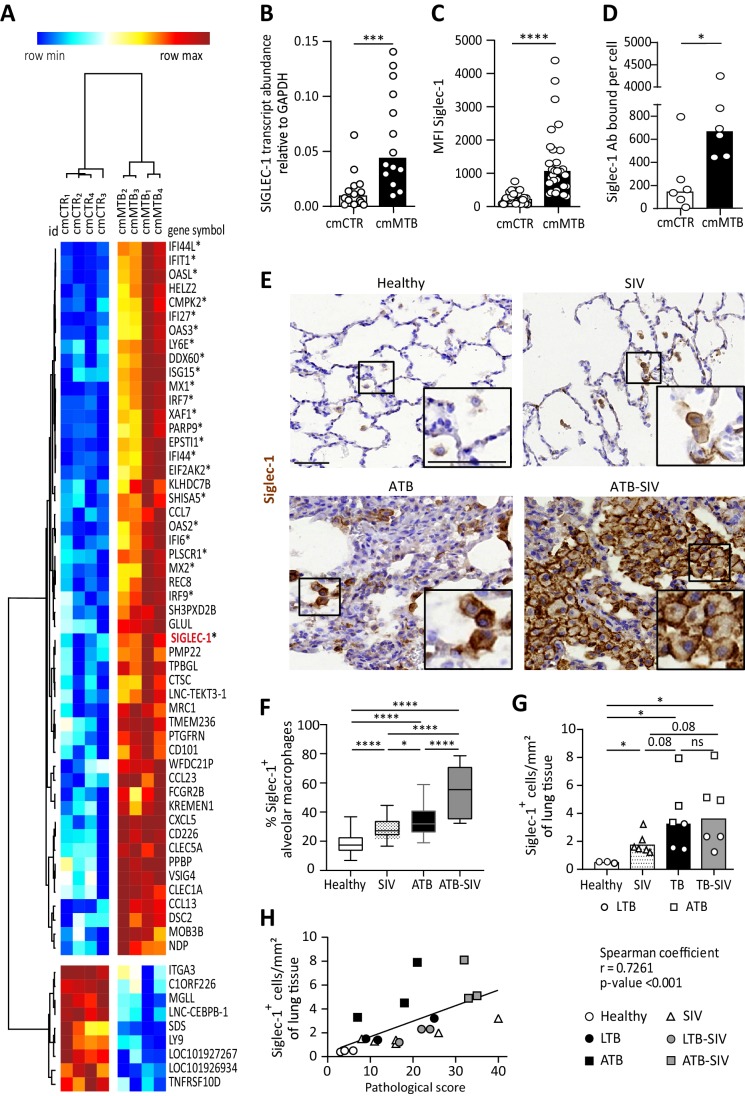
Tuberculosis-associated microenvironments induce Siglec-1 expression in macrophages. (**A–D**) For 3 days, human monocytes were differentiated into macrophages with cmCTR (white) or cmMTB (black) supernatants. (**A**) Heatmap from a transcriptomic analysis (GEO submission GSE139511) illustrating the top 60 differentially expressed genes (DEGs) between cmCTR- or cmMTB-cells. Selection of the top DEGs was performed using an adjusted p-value ≤ 0.05, a fold change of at least 2, and a minimal expression of 6 in a log_2_ scale. Hierarchical clustering was performed using the complete linkage method and the Pearson correlation metric with Morpheus (Broad Institute). Interferon-stimulated genes (ISG) are labelled with an asterisk and Siglec-1 is indicated in red. (**B–D**) Validation of Siglec-1 expression in cmMTB-treated macrophages. Vertical scatter plots showing the relative abundance to mRNA (**B**), median fluorescent intensity (MFI) (**C**), and mean number of Siglec-1 antibody binding sites per cell (**D**). Each circle represents a single donor and histograms median values. (**E**) Representative immunohistochemical images of Siglec-1 staining (brown) in lung biopsies of healthy, SIV infected (SIV), active TB (ATB), and co-infected (ATB-SIV) non-human primates (NHP). Scale bar, 100 µm. Insets are 2x zoom. (**F**) Vertical Box and Whisker plot indicating the distribution of the percentage of Siglec-1^+^ alveolar macrophages in lung biopsies from the indicated NHP groups. Quantification analysis from n = 800 alveolar macrophages grouped from three independent animals per NHP group. (**G**) Vertical scatter plots displaying the number of cells that are positive for Siglec-1 per mm² of lung biopsies from the indicated NHP groups. Each symbol represents a single animal per NHP group. (**H**) Correlation between Siglec-1^+^ cells per mm² of lung tissue and the pathological score for healthy (white circle), SIV^+^ (white triangles), latent (black circle) or active (black square) TB, and SIV^+^ with latent (grey circle) or active (grey square) TB. Each symbol represents a single animal per NHP group. Mean value is represented as a black line. Statistical analyses: Two-tailed, Wilcoxon signed-rank test (**B–D**), Mann-Whitney unpaired test (**F–G**), Spearman correlation (**H**). *p<0.05, **p<0.01, ***p<0.001, ****p<0.0001. ns: not significant. See Figure 1—source data 1. Figure 1—source data 1.Raw data and statistical analyses supporting Siglec-1 expression in human and non-human primate macrophages exposed to TB-associated microenvironment.

TB-induced M(IL-10) macrophages are highly susceptible to HIV-1 infection and spread (Souriant et al., 2019). To assess the global gene expression landscape in these cells, we performed a genome-wide transcriptome analysis (GEO submission GSE139511). To this end, we employed our published in vitro model (Lastrucci et al., 2015), which relies on the use of conditioned medium from either mock- (cmCTR) or Mtb-infected (cmMTB) human macrophages. As we described and observed before and herein, cmMTB-treated cells were positive for the M(IL-10) markers (CD16^+^CD163^+^MerTK^+^ and phosphorylated STAT3), and displayed a high rate of HIV-1 infection, as compared to those treated with cmCTR (Lastrucci et al., 2015). A distinct 60 gene-transcript signature was defined in cmMTB-treated cells, using a combination of the expression level, statistical filters and hierarchical clustering; 51 genes were up-regulated and nine genes were down-regulated in cmMTB- compared to cmCTR-treated cells (Figure 1A). We compared expression data of cmMTB- and cmCTR-treated cells to public genesets available from MSigDB (Broad Institute) using the gene set enrichment analysis (GSEA) algorithm (Subramanian et al., 2005). As shown in Figure 1—figure supplement 1A, a significant fraction of genes that were up-regulated in response to interferon (IFN) type I (*e.g.* IFNα) and II (*i.e.* IFNγ), were also found, as a group, significantly up-regulated in cmMTB-treated cells in comparison to control samples (FDR q-value:<10^-3^). IFN-stimulated genes (ISG) usually exert antiviral activities (McNab et al., 2015; Schneider et al., 2014) and cannot be inferred as obvious candidates to facilitate HIV-1 infection. However, among this ISG signature, the up-regulation of Siglec-1 (7.4-fold, adjusted p-value of 0.0162) in cmMTB-treated cells captured our attention due to its known role in HIV-1 pathogenesis (Izquierdo-Useros et al., 2014; O'Neill et al., 2013). We confirmed a high Siglec-1 expression in cmMTB-treated macrophages at the mRNA (Figure 1B), intracellular and cell-surface protein (Figure 1C and Figure 1—figure supplement 1B–D) levels. This effect was superior to the level obtained in HIV-1-infected cells (Figure 1—figure supplement 1E). Particularly, cmMTB-treated macrophages displayed high density of Siglec-1 surface expression applying a quantitative FACS assay that determines the absolute number of Siglec-1 antibody binding sites per cell (Figure 1D).

**Figure 1—figure supplement 1.**
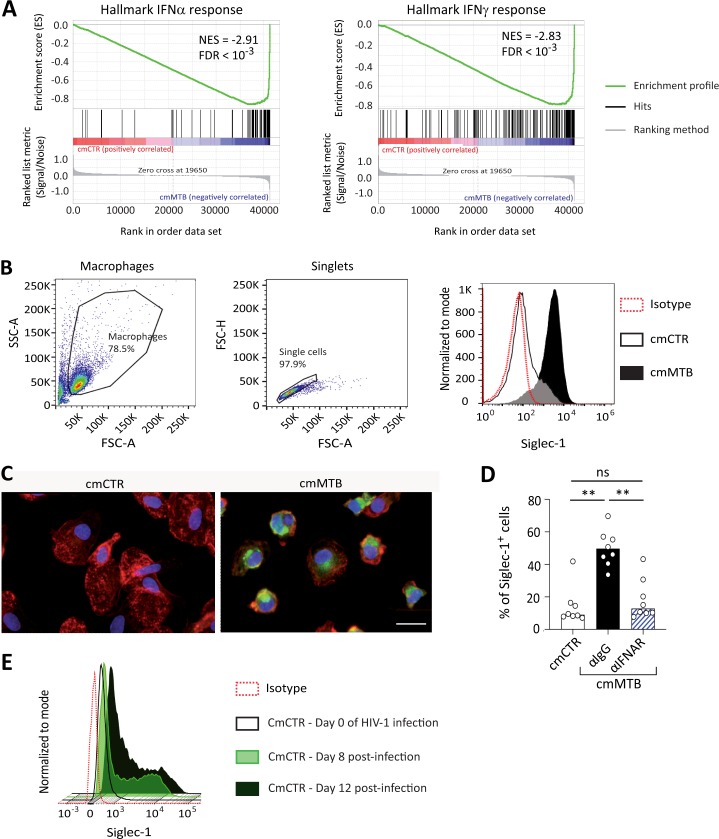
Tuberculosis-associated microenvironments increase Siglec-1 expression in human macrophages. (**A–D**) For 3 days, human monocytes were differentiated into macrophages with cmCTR (white) or cmMTB (black) supernatants. (**A**) (*Left*) Gene set enrichment plot of the interferon alpha (IFNα) response (hallmark collection of MSigDB). This plot shows the distribution of the barcode between macrophages exposed to cmCTR (red) *versus* cmMTB (blue) supernatants. Each bar of the barcode corresponds to a signature gene of the gene set. The skewing to the right indicates enrichment in macrophages exposed to cmMTB *versus* cmCTR supernatant of genes up-regulated in response to IFNα. (*Right*) Gene set enrichment plot of the IFNγ response (hallmark collection of MSigDB). (**B**) Flow cytometry gating strategy to assess Siglec-1 cell-surface expression in human macrophages exposed to cmCTR (white) and cmMTB (black). (*Left*) Based on size (FSC-A) and granularity (SSC-A), a gate was created to separate human macrophages from cell debris and dying cells. Macrophages were then subjected through a second gate based FSC Area and Height Scaling (FSC-A and FSC-H) to separate singlets from doublets. (*Right*) Based on the singlet gate, the histogram plot illustrates Siglec-1 expression that is higher in cmMTB- (black) than in cmCTR-treated (white) macrophages. (**C**) Representative immunofluorescence of Siglec-1 intracellular staining (green), actin (red) and nuclei (blue) after 3 days of monocyte conditioning with cmMTB. Scale bar, 10 μm. (**D**) Vertical scatter plot showing the quantification of Siglec-1 intracellular staining in cmCTR- or cmMTB-treated cells in the presence of an IFNAR-2 blocking (α-IFNAR) or control (α-IgG) antibodies during cell conditioning. Each circle represents a single donor and histograms median value. (**E**) Median fluorescence intensity (MFI) of Siglec-1 cell-surface expression in human macrophages exposed to cmCTR and infected with HIV-1 assessed by flow cytometry. The histogram plot illustrates Siglec-1 expression that increases within days post-HIV-1 infection of cmCTR-macrophages. Statistical analyses: Two-tailed, Wilcoxon signed-rank test (**D**). *p<0.05, **p<0.01, ns: not significant.

**Figure 1—figure supplement 2.**
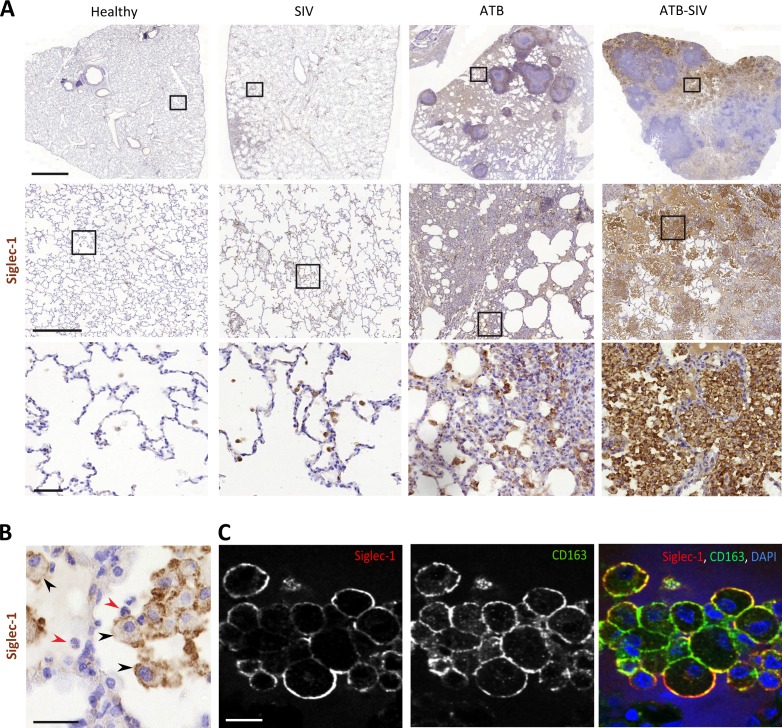
Tuberculosis-associated microenvironments increase Siglec-1 expression in non-human primate alveolar macrophages. (**A**) Accumulation of Siglec-1^+^ alveolar macrophages in the lung of co-infected non-human primates (NHP). Representative immunohistochemical images of Siglec-1 staining (brown) in lung biopsies of healthy, SIV-infected (SIV), active tuberculosis (ATB) and co-infected with SIV (ATB-SIV) NHP. Scale bars from top to bottom: 2 mm, 500 µm and 50 µm. (**B–C**) Siglec-1^+^ cells display the alveolar macrophage morphology. (**B**) Representative immunohistochemistry image from lung biopsy of an ATB-SIV NHP stained for Siglec-1 (brown). Siglec-1^+^ cells display a cell morphology with a single nucleus and large cytoplasm reminiscent of macrophage (black arrowhead); Siglec-1^-^ cells display a different nucleus morphology and small cytoplasm reminiscent of neutrophils (red arrowhead). Scale bar, 20 µm. (**C**) Representative immunofluorescence images of alveolar macrophages found in lung biopsy of a representative ATB-SIV NHP stained for Siglec-1 (red), CD163 (green) and nuclei (DAPI, blue). Scale bar, 20 µm.

These data indicate that Siglec-1 is highly expressed in human macrophages exposed to TB-associated microenvironments and potentially in the context of TB-HIV co-infection.

### Siglec-1^+^ alveolar macrophage abundance correlates with pathology in co-infected primates

NHP has been an invaluable in vivo model to better understand the role of macrophages in SIV/HIV pathogenesis (Merino et al., 2017). Considering Siglec-1 binds sialylated lipids present in the envelop of HIV-1 and SIV (Izquierdo-Useros et al., 2012a; Puryear et al., 2012), we examined the presence of Siglec-1 positive alveolar macrophages in lung biopsies obtained from different NHP groups: (i) co-infected with Mtb (active or latent TB) and SIV, (ii) mono-infected with Mtb (active or latent TB), (iii) mono-infected with SIV, and (iv) healthy (Supplementary file 1-Table S1, Figure 1—figure supplement 2A; Cai et al., 2015; Kuroda et al., 2018; Souriant et al., 2019). Histological immuno-staining confirmed the presence of Siglec-1^+^ alveolar macrophages in the lungs of healthy NHP (Figure 1E–F and Figure 1—figure supplement 2A), and revealed its significant increase in NHP mono-infected with either Mtb or SIV (Figure 1E–F and Figure 1—figure supplement 2A). Strikingly, we noticed a massive abundance of these cells in co-infected NHP (Figure 1E–F and Figure 1—figure supplement 2A). Concerning the overall abundance of Siglec-1^+^ leukocytes in lungs, we observed a significant increase in all infected NHP in comparison to healthy, with a higher tendency in active TB or co-infected NHP (Figure 1G and Figure 1—figure supplement 2A). In fact, the number of Siglec-1^+^ leukocytes correlated positively with the severity of NHP pathology (Figure 1H, Supplementary file 1-Table S2). Based on their cell morphology, localization in alveoli, and co-expression with the macrophage marker CD163 (Figure 1—figure supplement 2A–C), Siglec-1^+^ cells were identified as alveolar macrophages.

Collectively, these data show that Siglec-1 is up-regulated in alveolar macrophages in the context of a retroviral co-infection with active TB.

### Siglec-1 expression is dependent on Mtb-induced type I IFN signaling

**Figure 2.**
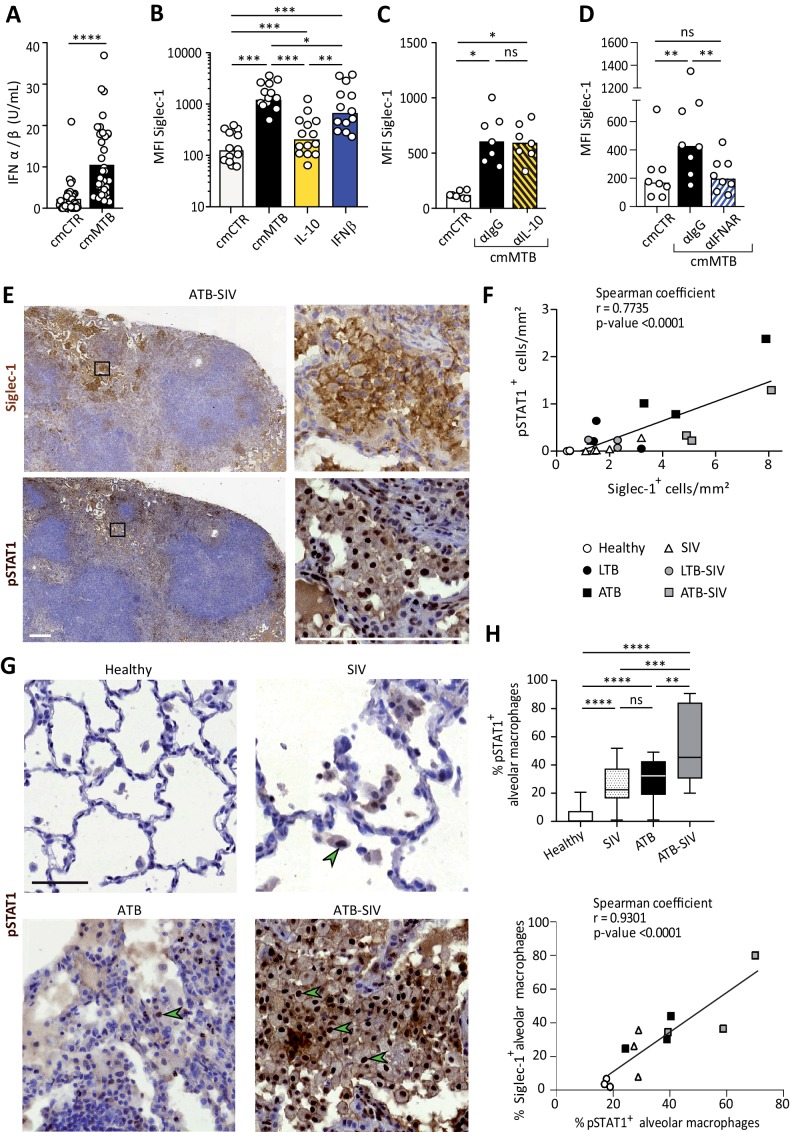
Siglec-1 expression is dependent on Mtb-induced type I IFN signaling. (**A**) Vertical scatter plot showing the relative abundance of IFN-I in cmCTR (white) and cmMTB (black) media, as measured indirectly after 24 hr exposure to the HEK-Blue IFN-α/β reporter cell line yielding reporter activity in units (U) per mL. (**B–D**) Vertical scatter plots displaying the median fluorescent intensity (MFI) of Siglec-1 cell-surface expression after three days of monocyte differentiation into macrophages either with cmMTB (black) or cmCTR (white), the indicated recombinant cytokines (**B**), the presence of an IL-10 depletion (α-IL-10) or a control (α-IgG) antibodies (**C**), or the presence of an IFNAR-2 blocking (α-IFNAR) or control (α-IgG) antibodies (**D**). (**E**) Representative serial immunohistochemical images of lung biopsies of a co-infected (ATB-SIV) NHP stained for Siglec-1 (brown, top) and pSTAT1 (brown, bottom). Scale bar, 250 µm. Insets are 10x zooms. (**F**) Correlation of the percentage of cells positive for Siglec-1 and pSTAT1, as measured per mm^2^ of lung tissue from the indicated NHP groups. Mean value is represented as a black line. (**G**) Representative immunohistochemical images of lung biopsies from the indicated NHP group stained for pSTAT1 (brown). Arrowheads show pSTAT1-positive nuclei. Scale bar, 500 µm. (**H**) Upper panel: Vertical Box and Whisker plot illustrating the percentage of pSTAT1^+^ alveolar macrophages in lung biopsies from the indicated NHP groups. Quantification analysis from n = 600 alveolar macrophages grouped from three independent animals per NHP group. Lower panel: Correlation of the percentage of alveolar macrophages positive for Siglec-1 and pSTAT1, from the indicated NHP groups. Mean value is represented as a black line. (**A–D**) Each circle within vertical scatter plots represents a single donor and histograms median value. Statistical analyses: Two-tailed, Wilcoxon signed-rank test (**A–D**), Spearman correlation (F, H lower panel), and Mann-Whitney unpaired test (H, upper panel). *p<0.05, **p<0.01, ***p<0.001, ****p<0.0001. ns: not significant. See Figure 2—source data 1. Figure 2—source data 1.Raw data and statistical analyses supporting that IFN-I induced by M. tuberculosis is responsible for Siglec-1 expression in human and non-human primate macrophages.

Siglec-1 is an ISG whose expression is induced by IFN-I in myeloid cells (Hartnell et al., 2001). In addition to viral infection, IFN-I is also induced in TB and known to mainly play a detrimental role (McNab et al., 2015; Moreira-Teixeira et al., 2018). Siglec-1 expression has not been described in the TB context or in co-infection with retroviruses such as SIV or HIV-1, therefore we assessed whether IFN-I stimulates Siglec-1 expression in TB-associated microenvironments. First, we found that cmMTB contains high amounts of IFN-I compared to cmCTR (Figure 2A). Next, we showed that recombinant IFN-β significantly increased Siglec-1 cell-surface expression in macrophages, close to the level induced by cmMTB (Figure 2B). Interestingly, we observed a modest, albeit significant, induction of Siglec-1 expression in cells treated with interleukin 10 (IL-10), a cytokine we have previously shown to be abundant in cmMTB (Lastrucci et al., 2015) and that renders macrophages highly susceptible to HIV-1 infection (Souriant et al., 2019). However, IL-10 depletion had no effect on Siglec-1 expression by cmMTB-treated cells (Figure 2C). By contrast, blocking the IFN-I receptor (IFNAR-2) during cmMTB treatment fully abolished the expression of Siglec-1 (Figure 2D and Figure 1—figure supplement 1D), indicating that IFN-I is the responsible factor for Siglec-1 up-regulation in cmMTB-treated cells.

IFN-I binding to IFNAR leads to the phosphorylation and nuclear translocation of the transcription factor STAT1, whose role is essential for transcription of ISG (Ivashkiv and Donlin, 2014). We thus examined the status of STAT1 activation in co-infected NHP lung tissue. Histological staining of serial sections of co-infected lungs revealed that zones rich in Siglec-1^+^ leukocytes also exhibited positivity for nuclear phosphorylated STAT1 (pSTAT1) (Figure 2E), and the abundance of these two markers strongly correlated with the active status of TB in the different NHP groups (Figure 2F). Moreover, we found that the majority of Siglec-1^+^ alveolar macrophages were also positive for nuclear pSTAT1 in the infected NHP groups compared to healthy (Figure 2E and G). In fact, there was a higher number of pSTAT1^+^ alveolar macrophages in TB-SIV co-infected lungs when compared to those from mono-infected NHP and this number directly correlates with the number of Siglec-1^+^ alveolar macrophages (Figure 2G–H).

Altogether, these data demonstrate that Siglec-1 expression in human macrophages is controlled by IFN-I in a TB-associated microenvironment, and suggest the involvement of the IFN-I/STAT1/Siglec-1 axis in the pathogenesis of TB and co-infection with retroviruses.

### Siglec-1 localization on thick TNT is associated with their length and HIV-1 cargo

**Figure 3.**
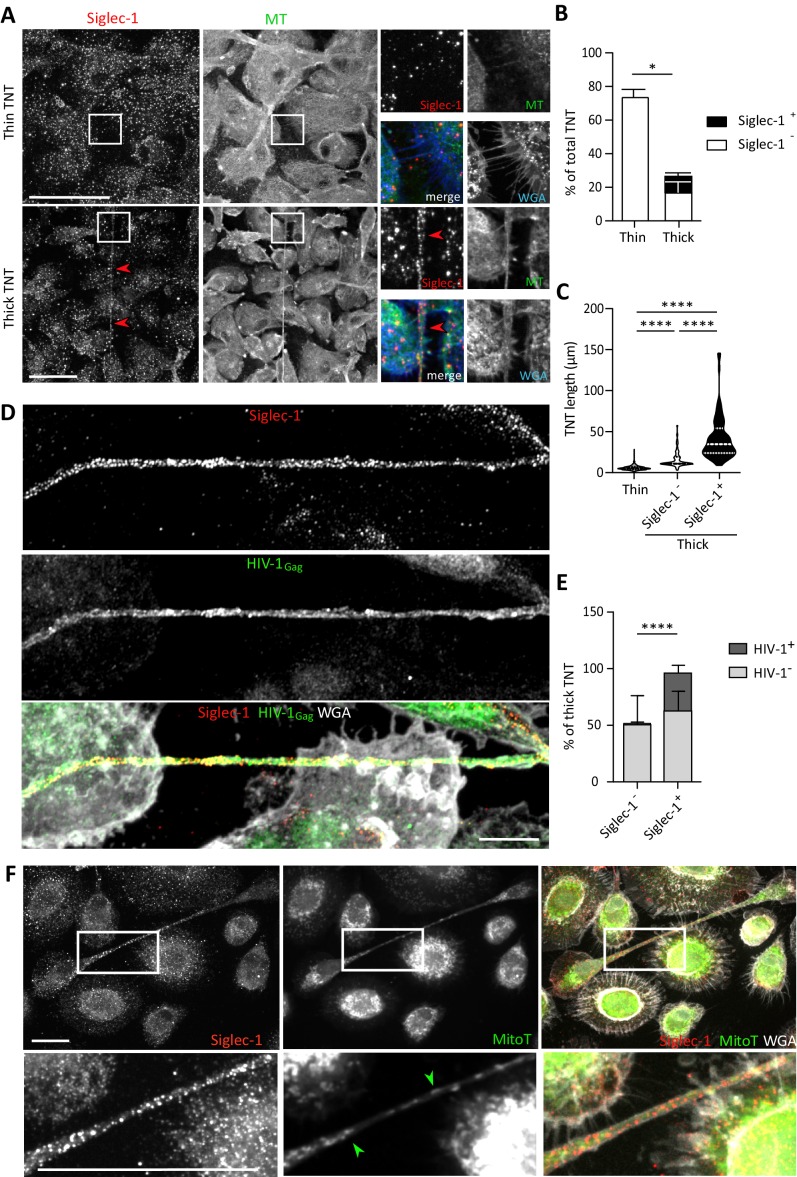
Siglec-1 localization on thick TNT is associated with their length and HIV-1/mitochondria cargo. (**A–F**) Human monocytes were differentiated into macrophages with cmMTB for 3 days, and then infected with HIV-1-ADA strain (unless indicated otherwise) and fixed 3 days post-infection. (**A**) Representative immunofluorescence images of cmMTB-treated macrophages infected with HIV-1-ADA, and stained for extracellular Siglec-1 (red), intracellular tubulin (MT, green) and Wheat Germ Agglutinin (WGA, blue). Inserts are 3x zooms. Red arrowheads show Siglec-1 localization on TNT. Scale bar, 20 µm. (**B**) Vertical bar plot showing the semi-automatic quantification of Siglec-1^+^ TNT (black) and Siglec-1^-^ TNT (white) in thick (WGA^+^, MT^+^) and thin (WGA^+^, MT^-^) TNT. 400 TNT were analyzed from two independent donors. (**C**) Siglec-1^+^ TNT exhibit a larger length index. Violin plots displaying the semi-automatic quantification of TNT length (in μm) for thin (WGA^+^, MT^-^), and thick TNT (WGA^+^, MT^+^) expressing Siglec-1 or not. 400 TNT were analyzed per condition from two independent donors. (**D**) Representative immunofluorescence images of cmMTB-treated macrophages 3 day post-infection with HIV-1-NLAD8-VSVG strain, and stained for extracellular Siglec-1 (red), intracellular HIV-1_Gag_ (green) and WGA (grey). Scale bar, 10 µm. (**E**) Vertical bar plots indicating the quantification of presence (dark grey) or absence (light grey) of HIV-1_Gag_ in thick TNT (WGA^+^, MT^+^) expressing Siglec-1 or not. 120 TNT in at least 1000 cells were analyzed from four independent donors. (**F**) Representative immunofluorescence images of cmMTB-treated macrophages infected with HIV-1-ADA loaded with MitoTracker (MitoT, green), and stained for extracellular Siglec-1 (red) and WGA (grey). Inserts are 3x zooms. Green arrowheads show mitochondria inside TNT. Scale bar, 10 µm. Statistical analyses: Two-way ANOVA comparing the presence of Siglec-1 in thin and thick TNT (**B**), and two-tailed Mann-Whitney unpaired test comparing TNT length (**C**) and the presence of HIV-1 in TNT (**E**). *p<0.05, ****p<0.0001. See Figure 3—source data 1. Figure 3—source data 1.Raw data and statistical analyses supporting Siglec-1 expression on thick TNT and its correlation with TNT length.

TNT formation is responsible for the increase in HIV-1 spread between human macrophages in TB-associated microenvironments (Souriant et al., 2019). To investigate whether Siglec-1 expression is involved in this process, we first examined its localization in the context of TNT formed by cmMTB-treated cells infected by HIV-1. We observed that Siglec-1 is localized mainly on microtubule (MT)-positive thick TNT, and not on thin TNT (Figure 3A and Figure 3—video 1). Semi-automatic quantification of hundreds of TNT showed that about 50% of thick TNT were positive for Siglec-1 (Figure 3B and Figure 3—figure supplement 1A). These TNT exhibited a greater length compared to those lacking Siglec-1 (Figure 3C). Importantly, unlike thin TNT, HIV-1 viral proteins are found mainly inside Siglec-1^+^ thick TNT (Figure 3D–E and Figure 3—video 2). In addition, these thick TNT also contained large organelles such as mitochondria (Figure 3F and Figure 3—figure supplement 1B), another characteristic distinguishing thick from thin TNT (Dupont et al., 2018; Onfelt et al., 2006). In general, we also noticed that the incidence of Siglec-1^+^ thick TNT between HIV-1 infected macrophages persisted for more than one week upon HIV-1 infection (Figure 3—figure supplement 1C), suggesting a high degree of stability for these TNT.

**Figure 3—figure supplement 1.**
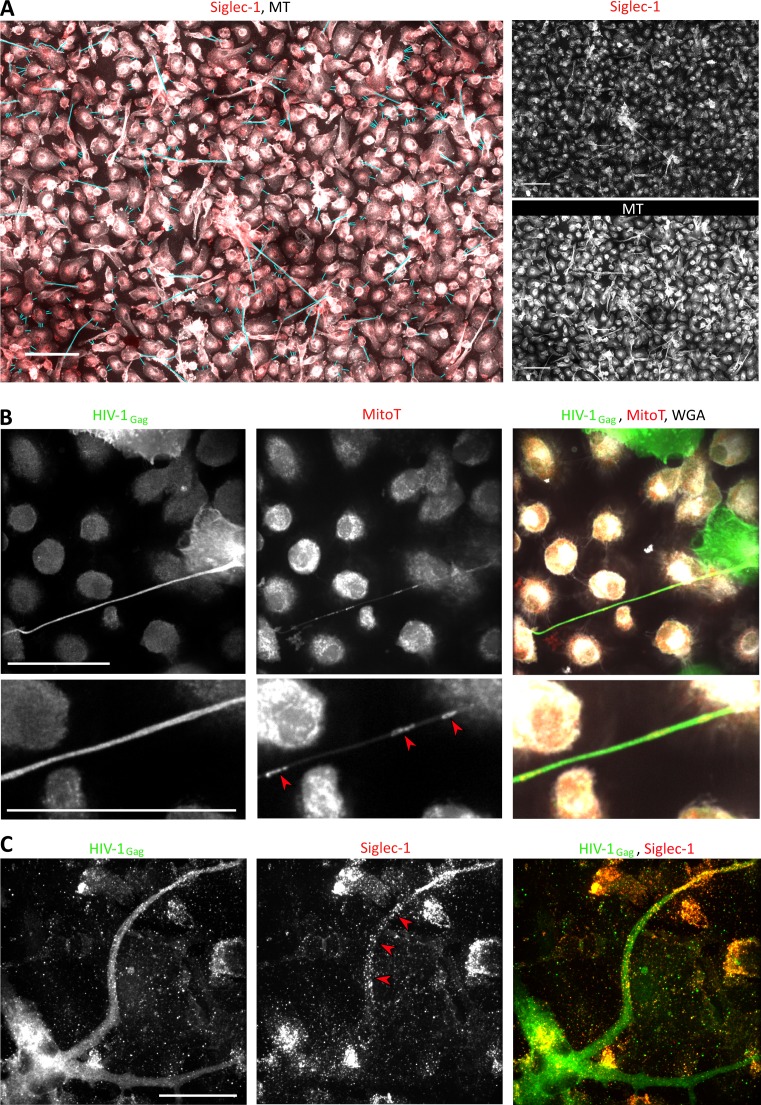
Siglec-1 localizes specifically on thick tunneling nanotubes that contain HIV-1_Gag_ and mitochondria. (**A–C**) Human monocytes were differentiated into macrophages with cmMTB for 3 days, infected with HIV-1-ADA strain (unless indicated otherwise) and then fixed at day 3 (**A–B**) or 14 (**C**) post-infection. (**A**) Representative immunofluorescence images used for semi-automatic quantification of TNT in cmMTB-treated macrophages infected with HIV-1. Cells were stained for extracellular Siglec-1 (red), intracellular tubulin (MT, grey) and Wheat Germ Agglutinin (WGA, not shown). Blue lines show all TNT considered. Thick (WGA^+^, MT^+^) and thin (WGA^+^, MT^-^) TNT were assessed for Siglec-1 positivity by applying a threshold and measured in length. Scale bar, 20 µm. (**B**) Representative immunofluorescence images of cmMTB-treated macrophages infected with HIV-1-NLAD8-VSVG, loaded with Mitotracker (MitoT, red) and stained for intracellular HIV-1_Gag_ (green) and WGA (grey). Red arrowheads show mitochondria inside HIV-1_Gag_-containing TNT. Inserts are 2x zoom. Scale bar, 20 µm. (**C**) Representative immunofluorescence images of cmMTB-treated macrophages infected with HIV-1 and kept in culture until day 14. Cells were fixed and stained for intracellular HIV-1_Gag_ (green), extracellular Siglec-1 (red) and WGA (grey). Red arrowheads show Siglec-1 on HIV-1_Gag_-containing TNT emanating from an infected multinucleated giant cell (MGC). Scale bar, 20 µm.

**Figure 3—video 1.** Related to Figure 3A. Z-stack of confocal microscopy images, showing Siglec-1 (red), microtubules (MT, green) and F-actin (grey) of day 6 HIV-1-infected macrophages, treated with cmMTB. Siglec-1 localizes on the thick MT^+^ F-actin^+^ TNT but not on thin MT^-^ F-actin^+^ TNT.

**Figure 3—video 2.** Related to Figure 3D. 3D reconstitution of confocal images, showing Siglec-1 (red), HIV-1_Gag_ (green) and WGA (grey) of day 6 HIV-1-infected macrophages, treated with cmMTB.

These findings reveal a strong localization of Siglec-1 on MT-positive thick TNT that correlates positively with a greater length and high cargo of HIV-1 or mitochondria, arguing for a functional capacity of Siglec-1^+^ TNT to transfer material to recipient cells over long distances.

### The Mtb-induced exacerbation of HIV-1 infection and spread in macrophages requires Siglec-1

**Figure 4.**
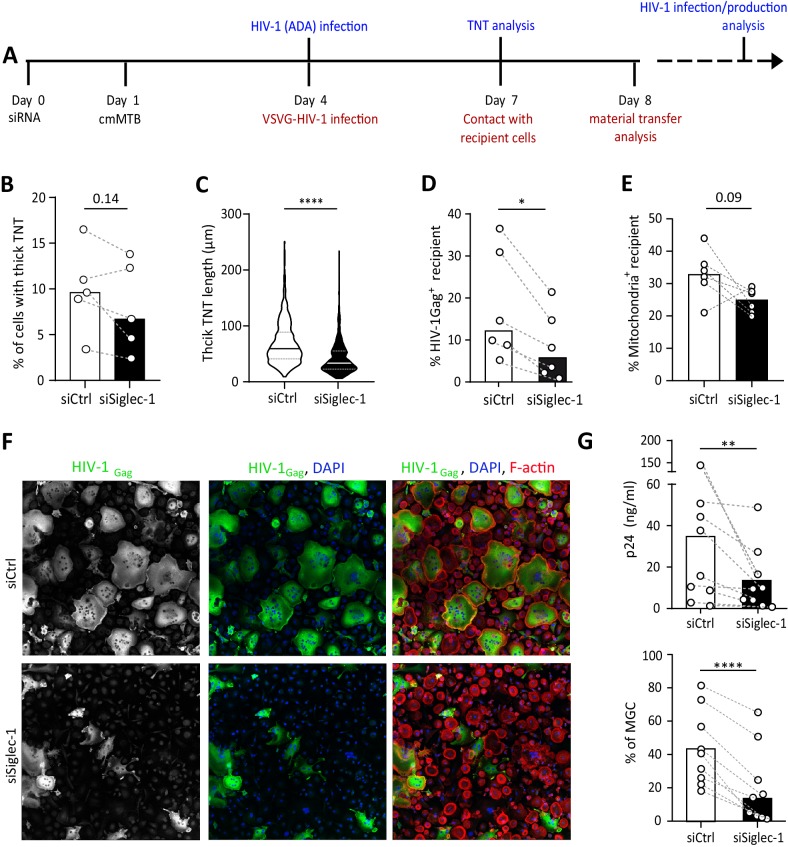
The exacerbation of HIV-1 infection and spread in macrophages treated with cmMTB requires Siglec-1. (**A**) Experimental design. Monocytes from healthy subjects were transfected with siRNA targeting of Siglec-1 (siSiglec-1, black) or not (siCtrl, white). A day after, monocytes were differentiated into macrophages with cmMTB for 3 days. Cells were then infected with HIV-1-ADA (blue protocol) to measure the formation (**B**) and length (**C**) of TNT at day 7, or assess HIV-1 production and multinucleated giant cell (MGC) formation at day 14 (**F–G**). In parallel, cells were either infected with HIV-NLAD8-VSVG or labelled with mitoTracker to measure the transfer (red protocol) of HIV-1 (**D**) or mitochondria (**E**), respectively. (**B**) Before-and-after plots showing the percentage of cells forming thick TNT (F-actin^+^, WGA^+^, MT^+^). (**C**) Violin plots displaying the semi-automatic quantification of TNT length (in μm) for thick (WGA^+^, MT^+^) TNT; 300 TNT were analyzed per condition from two independent donors. (**D–E**) Before-and-after plots indicating the percentage of HIV-1_Gag_^+^ cells (**D**) or MitoTracker^+^ cells (**E**) among CellTracker^+^ cells after 24 hr co-culture. (**F**) Representative immunofluorescence images of siRNA transfected cells treated with cmMTB, 14 days post-HIV-1 infection. Cells were stained for intracellular HIV-1_Gag_ (green), F-actin (red) and DAPI (blue). Scale bar, 500 µm. (**G**) Vertical scatter plots showing HIV-1-p24 concentration in cell supernatants (upper panel) and percentage of MGC (lower panel) at day 14 post-HIV-1 infection in cells represented in F (siSiglec-1, black; siCtrl, white). (**B, D, E and G**) Each circle represents a single donor and histograms median value. Statistical analyses: Paired t-test (B, G lower panel) or two-tailed, Wilcoxon signed-rank test (C-E, G upper panel). *p<0.05, **p<0.01, ****p<0.0001. See Figure 4—source data 1. Figure 4—source data 1.Raw data and statistical analyses supporting a role for Siglec-1 in TNT length, HIV-1 and mitochondrial cell-to-cell trasfer, and exacerbation of HIV-1 infection.

To evaluate a functional role for Siglec-1 in the susceptibility of macrophages to HIV-1 infection and spread induced by TB, Siglec-1 was depleted in cmMTB-treated cells by siRNA-mediated gene silencing (Figure 4A and Figure 4—figure supplement 1A). While this depletion did not affect the total number of thick TNT (Figure 4B and Figure 4—figure supplement 1B), we observed a 2-fold shortening of thick TNT in cells lacking Siglec-1 when compared to control cells (Figure 4C). Then, we performed a viral uptake assay in these cells using HIV-1-Gag-eGFP virus-like particles (GFP VLP) lacking the viral envelope glycoprotein but bearing sialylated lipids that interact with Siglec-1 on myeloid cells (Izquierdo-Useros et al., 2012b; Puryear et al., 2013). We consistently observed binding of VLP along Siglec-1^+^ thick TNT (Figure 4—figure supplement 1C). Yet, in the absence of Siglec-1, we noticed a significant reduction of VLP binding in comparison to control cells (Figure 4—figure supplement 1D). We confirmed this functional observation using a blocking monoclonal antibody against Siglec-1, showing that this receptor is involved in HIV-1 binding in cmMTB-treated cells (Figure 4—figure supplement 1E).

**Figure 4—figure supplement 1.**
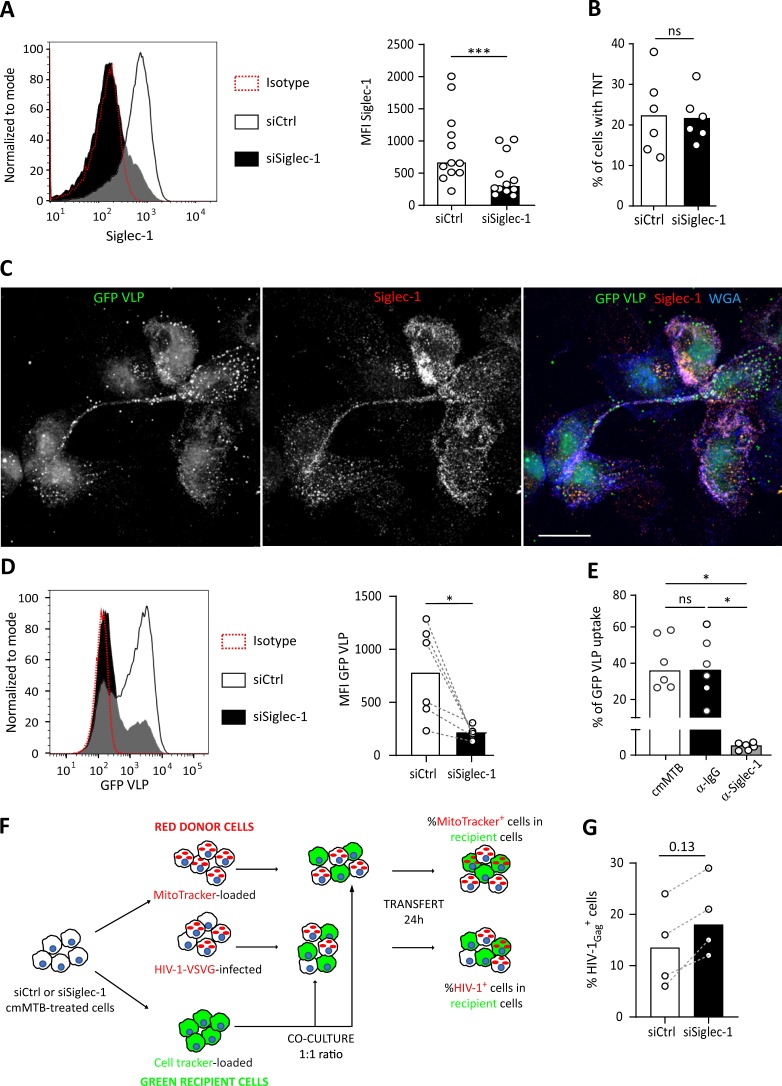
Siglec-1 is required for the capture and transfer of HIV-1 in cmMTB-treated macrophages. (**A–D, F–G**) Monocytes from healthy subjects were transfected with siRNA targeting of Siglec-1 (siSiglec-1, black) or not (siCtrl, white). A day after, monocytes were differentiated into macrophages with cmMTB for 3 days. (**A**) Representative histogram (*left*) and vertical scatter plot showing the median fluorescent intensity (MFI) (*right*) of Siglec-1 expression on the indicated cell populations. (**B**) Vertical scatter plot indicating the percentage of cells forming TNT. (**C–E**) Inhibition of Siglec-1 reduces binding of HIV-1-Gag−eGFP virus like particles (GFP-VLP). (**C**) Representative immunofluorescence images of cmMTB-treated cells incubated with GFP-VLP (green) for 3.5 hr. Cells were fixed and stained for extracellular Siglec-1 (red) and Wheat Germ Agglutinin (WGA, blue). Scale bar, 500 µm. (**D**) Representative histogram (*left*) and vertical scatter plot showing the median fluorescent intensity (MFI) (*right*) displaying of GFP-VLP binding in the indicated cell populations. (**E**) Vertical scatter plot showing the percentage of GFP-VLP binding in cmMTB treated cells pre-incubated with specific anti-Siglec-1 (α-Siglec-1, grey), anti-Isotype control antibodies (α-IgG, black) or mock (white). (**F**) Schematics of the experimental procedure for material (HIV-1 and mitochondria) transfer experiments. (**G**) Vertical scatter plot showing the percentage of HIV-1_Gag_^+^ cells at the time of co-culture experiment in the indicated cells. Statistical analyses: Two-tailed, Wilcoxon matched-pairs signed rank test (A, B, D-E, G). *p<0.05, ***p<0.001. ns: not significant. Figure 4—figure supplement 1—source data 1.Supplemental raw data and statistical analyses supporting the functional role of Siglec-1 in human macrophages using an siRNA-mediated gene silencing approach.

We then assessed the role of Siglec-1 in HIV-1 transfer between macrophages, as this receptor is also important for the transfer of the virus to CD4^+^ T cells (Akiyama et al., 2015; Izquierdo-Useros et al., 2012a; Puryear et al., 2013). We used an established co-culture system between cmMTB-treated macrophages that allows the transfer of the viral Gag protein from infected (donor, Gag^+^, red) to uninfected (recipient, CellTracker^+^, green) cells over 24 hr (Souriant et al., 2019; Figure 4—figure supplement 1F). Of note, since Siglec-1 facilitates the infection of macrophages (Zou et al., 2011), we used VSV-G pseudotyped viruses to avoid any effect on HIV-1 primo-infection. Like this, we ensured the viral content was equal in cells at the time of the co-culture despite the loss of Siglec-1 (Figure 4—figure supplement 1G). The siRNA-mediated depletion of Siglec-1 significantly diminished the capacity of cmMTB-treated macrophages to transfer HIV-1 to recipient cells (Figure 4D), indicating that this receptor is involved in the macrophage-to-macrophage viral spread (Souriant et al., 2019). Intriguingly, there was a decreasing tendency for the capacity of Siglec-1-depleted cmMTB-treated macrophages to transfer mitochondria to recipient cells compared to controls (Figure 4E and Figure 4—figure supplement 1F), alluding to a possible defect in mechanisms involved in intercellular material transfer including through thick TNT (Torralba et al., 2016). Remarkably, using replicative HIV-1 ADA strain (Figure 4A), we showed that silencing Siglec-1 expression in cmMTB-treated cells abolished the exacerbation of HIV-1 infection and production, as well as the enhanced formation of multinucleated giant cells (MGC) (Figure 4F–G), which are pathological hallmarks of HIV-1 infection of macrophages (Vérollet et al., 2015; Vérollet et al., 2010).

These results determine that TB-induced Siglec-1 expression plays a key part in HIV-1 uptake and efficient cell-to-cell transfer, resulting in the exacerbation of HIV-1 infection and production in M(IL-10) macrophages.

## Discussion

In this study, we investigated potential mechanisms by which Mtb exacerbates HIV-1 infection in macrophages, and uncovered a deleterious role for Siglec-1 in this process. These findings have different contributions to our understanding of this receptor in the synergy between Mtb and distinct retroviral infections, and also for TNT biology in host-pathogen interactions.

Our global transcriptomic approach revealed the up-regulation of Siglec-1, as part of an ISG-signature enhanced in macrophages exposed to a TB-associated microenvironment. Although pulmonary active TB has been characterized as an IFN-I-driven disease (Berry et al., 2010; McNab et al., 2015; Moreira-Teixeira et al., 2018), there are no report in the literature about a role for Siglec-1 in TB or in Mtb co-infection with retroviruses. Expression of Siglec-1 is restricted to myeloid cells except circulating monocytes (Crocker et al., 2007), and is enhanced by IFN-I (Puryear et al., 2013; Rempel et al., 2008) and during HIV-1 infection (Pino et al., 2015). In addition, human alveolar macrophages are distinguished from lung interstitial macrophages by Siglec-1 expression (Yu et al., 2016). In this study, we determined that IFN-I present in TB-associated environment is responsible for Siglec-1 overexpression in human macrophages, which resembled that obtained in HIV-1-infected cells. While we saw a modest induction of Siglec-1 in macrophages upon IL-10 treatment, its depletion from the TB-associated microenvironment had no effect on Siglec-1 expression. This could be explained by the fact that IL-10 induces the autocrine production of IFN-I (Ziegler-Heitbrock et al., 2003) to indirectly modulate Siglec-1 expression in M(IL-10) macrophages, which then contributes to the exacerbation of HIV-1 infection as we previously reported (Souriant et al., 2019). In the context of the most closely related lentivirus to HIV, namely SIV, we not only confirmed the presence of Siglec-1^+^ alveolar macrophages in SIV-infected NHP, but also reported the high abundance of these cells in active TB and in co-infected NHP groups, when compared to healthy ones. Importantly, we associated the high abundance of Siglec-1^+^ leukocytes with the increase NHP pathological scores, and it correlated positively to the detection of pSTAT1^+^ macrophage nuclei in histological staining of serial sections of lung biopsies from co-infected NHP. This is in line with a recent report on the presence of IFN-I, IFNAR and different ISG in alveolar and lung interstitial tissue from NHP with active TB (Mattila, 2019), and with the fact that the in vivo expression of Siglec-1 is up-regulated early in myeloid cells after SIV infection and maintained thereafter in the pathogenic NHP model (Jaroenpool et al., 2007). In TB-SIV co-infection, we hypothesized that IFN-I is not exerting the expected antiviral effect, but instead is concomitant with chronic immune activation and attenuated by the high expression of Siglec-1 in myeloid cells, as recently proposed in the HIV-1 context (Akiyama et al., 2017). Altogether, these findings uncover the IFN-I/STAT1/Siglec-1 axis as a mechanism established by Mtb to exacerbate HIV-1 infection in myeloid cells, and call for the need to further investigate this signaling pathway in TB pathogenesis.

Another aspect worth highlighting is the impact that Siglec-1 expression has in the capture and transfer of HIV-1 by M(IL-10) macrophages, in particular in the context of TNT. First, we reported that Siglec-1 is located on MT-positive thick (and not on thin) TNT, correlating positively with increased length and HIV-1 cargo. To our knowledge, no receptor has been described so far to be present mainly on thick TNT, making Siglec-1 an unprecedented potential marker for this subtype of TNT (Dupont et al., 2018). Second, viral uptake assays demonstrated the functional capacity of Siglec-1, including on thick TNT, to interact with viral-like particles bearing sialylated lipids; loss-of-function approaches showed Siglec-1 is important in the capture of these viral particles. Third, Siglec-1 depletion correlated with a decrease in thick TNT length, but had no effect in the total number of thick TNT. This suggests that, while the IFN-I/STAT1 axis is responsible for Siglec-1 expression in M(IL-10) macrophages, it does not contribute to TNT formation. This is line with our previous report where TNT formation induced by TB-associated microenvironments depended on the IL-10/STAT3 axis (Souriant et al., 2019). Concerning the shortening of thick TNT length, we infer that it may reflect a fragile state due to an altered cell membrane composition in the absence of Siglec-1; TNT are known for their fragility towards light exposure, shearing force and chemical fixation (Rustom et al., 2004). We hypothesize that the longer the TNT is, the more rigidity it requires to be stabilized. Cholesterol and lipids are known to increase membrane rigidity (Redondo-Morata et al., 2012) and are thought to be critical for TNT stability (Lokar et al., 2012; Thayanithy et al., 2014). Thus, the presence of Siglec-1 in thick TNT may affect the cholesterol and lipid composition *via* the recruitment of GM1/GM3 glycosphingolipid-enriched microvesicles (Halász et al., 2018). In fact, TNT formation depends on GM1/GM3 ganglioside and cholesterol content (Kabaso et al., 2011; Lokar et al., 2012; Osteikoetxea-Molnár et al., 2016; Tóth et al., 2017). Since GM1 and GM3 glycosphingolipids are *bona fide* ligands for Siglec-1 (Puryear et al., 2013), it is likely that Siglec-1^+^ thick TNT exhibit a higher lipid and cholesterol content, and hence an increase of membrane rigidity that favors the stability of longer TNT. Fourth, Siglec-1-depleted donor macrophages were less capable to transfer HIV-1, and to some extend mitochondria, to recipient cells. While infectious synapse and exososome release are mechanisms attributed to Siglec-1 that contribute to cell-to-cell transfer of HIV-1 (Bracq et al., 2018; Gummuluru et al., 2014; Izquierdo-Useros et al., 2014), they accomplish so extracellularly. Here, we speculate that Siglec-1 participates indirectly in the intracellular HIV-1 transfer *via* TNT as a tunnel over long distance, suggesting that factors affecting TNT rigidity favor distal viral dissemination while ensuring protection against immune detection. Independent of HIV-1 infection, we also noticed that cmMTB-treated cells depleted for Siglec-1 displayed a decreasing tendency to transfer mitochondria among them. As TNT-transferred mitochondria are known to alter the metabolism and functional properties of recipient cells under steady state conditions or in the cancer context, it implies that Siglec-1 may also influence key metabolic pathways such as glycolysis, pentose phosphate and lipid metabolism, among others (Hekmatshoar et al., 2018). This is important because, for example, the gain of cancer drug resistance is directly associated to TNT-mediated mitochondria transfer, thus Siglec-1 may represent a novel therapeutic strategy to overcome cancer cell drug resistance. Finally, the depletion of Siglec-1 abrogated the exacerbation of HIV-1 infection and production induced by TB in M(IL-10) macrophages. This is likely to result from an accumulative effect of deficient capture and transfer of HIV-1 in the absence of Siglec-1. However, these results do not discern the specific contribution of Siglec-1 to the cell-to-cell transmission of HIV-1 *via* TNT from that obtained through other mechanisms (Bracq et al., 2018). Future studies will address whether the contribution of Siglec-1 to cell-to-cell transfer mechanisms has an impact in Mtb dissemination (Onfelt et al., 2006).

In conclusion, our study identifies Siglec-1 as a key factor involved in the exacerbation of HIV-1 infection in macrophages conditioned by cmMTB. It is worth noting that we have previously reported that a loss-of-function variant in Siglec-1 in the human population does not conclusively establish a role for this receptor in AIDS progression, even though ex vivo experiments demonstrated that cells from these individuals were functionally null or partially defective for Siglec-1 expression along with poor HIV-1 capture and transmission (Martinez-Picado et al., 2016). While this may be counterintuitive for proposing Siglec-1 as a therapeutic target to limit viral dissemination in the co-infection context, there are several challenges to the study of Siglec-1 variants, such as limited cohort size, the lack of complete clinical records, and the restriction to study only off-therapy periods, among others (Martinez-Picado et al., 2017). Therefore, there is a strong need for future work targeting Siglec-1 to unveil its in vivo contribution of the mononuclear phagocyte system to HIV-1 pathogenesis to fully exploit the therapeutic potential of this receptor. Beyond the infectious disease context, our study also sheds light on a new homeostatic function for Siglec-1 in human macrophages, such as intercellular communication facilitated by TNT. We argue that Siglec-1 localization on thick TNT has a physiological significance to macrophage biology in health and disease.

## Materials and methods

**Key resources table:** 

Reagent type (species) or resource	Designation	Source or reference	Identifiers	Additional information
*M. tuberculosis*	H37Rv	*Derived from E.R. Baldwin's human-lung isolate H37 by W. Steenken New York, United States, 1934*	(ATCC 25618)	
HIV-1	ADA	Gift from Dr. S Benichou Institut Cochin, Paris, France	N/A	
HIV-1	NLAD8	Gift from Dr. S Benichou Institut Cochin, Paris, France	N/A	
HIV-1	ADA Gag-iGFP-VSVG	This paper	This paper	
Buffy Coat	Leukocytes	Etablissement Français du Sang, Toulouse, France	N/A	
Lung biopsies from rhesus macaques	Histological slides	Tulane National Primate Research Center	N/A	
Cell line (human HeLa JC.53)	TZM-bl	NIH AIDS Reagent Program	Cat# 8129	Cultured in: DMEM, 90%; FBS, 10%; 100 units of Penicillin and 0.1 mg/mL of Streptomycin
Cell line (human)	HEK-293T	NIH AIDS Reagent Program	Cat# 3318	Cultured in: DMEM, 10% FCS
Cell line (human)	HEK-Blue IFN-α/β Cells	Invivogen	Cat# hkb-ifnab	Cultured in: DMEM, 4.5 g/l glucose, 2 mM L-glutamine, 10% (v/v) heat-inactivated fetal bovine serum, 100 U/ml penicillin, 100 µg/mL streptomycin, 100 µg/mL Normocin
Transfected construct (human)	siRNA to Siglec-1 (SMART-Pool)	Horizon Discovery	Cat# L-017521-01-0020	(200 nM)
Transfected construct (human)	siRNA scramble (SMART Pool)	Horizon Discovery	Cat# D-001810-10-50	(200 nM)
Antibody	Mouse monoclonal anti‑human Siglec-1 (clone 7-293)	Biolegend	Cat# 346008 RRID:AB_11147948	FACS (1 µg/mL)
Antibody	Mouse monoclonal anti‑human CD16 (clone 3G8)	Biolegend	Cat# 302019 and 302018; RRID:AB_492974 andAB_314218	FACS (1 µg/mL)
Antibody	Mouse monoclonal anti‑human CD163 (clone GHI/61)	Biolegend	Cat# 333608 RRID:AB_2228986	FACS (1 µg/mL)
Antibody	Mouse monoclonal anti‑human MerTK (clone 590H11G1E3)	Biolegend	Cat# 367607 RRID:AB_2566400	FACS (1 µg/mL)
Antibody	Rabbit monoclonal anti‑human STAT1 (clone 42H3)	Cell Signaling Technology	Cat# 9175 RRID:AB_2197984	WB (1:100)
Antibody	Rabbit anti‑human actin (a.a. 20‑33)	Sigma‑Aldrich	Cat# A5060 RRID:AB_476738	WB (1:100)
Antibody	Rabbit polyclonal anti-a-tubulin	Abcam	Cat# ab18251 RRID:AB_2210057	IF (5 µg/mL)
Antibody	Mouse monoclonal anti-Siglec-1 (clone hsn 7D2)	Novus Biologicals	Cat# NB 600-534 RRID:AB_526814	IF (10 µg/mL) IHC (1:200)
Antibody	Mouse monoclonal anti-Gag RD1 (clone KC57)	NIH AIDS Reagent program	Cat# 13449	IF (1:200)
Antibody	Mouse monoclonal anti-HIV-1 p24 (clone 183-H12-5C)	NIH AIDS Reagent Program	Cat# 3537	ELISA (2.5 µg/mL)
Antibody	Human polyclonal anti-HIV Immune Globulin (HIVIG)	NIH AIDS Reagent Program	Cat# 3957	ELISA (6.25 µg/mL)
Antibody	Polyclonal goat anti-human IgG	Sigma-Aldrich	Cat# A0170	ELISA (1:10000)
Antibody	Mouse monoclonal anti‑human CD163 (clone 10D6)	Leica/Novocastra	Cat# NCL-L-CD163 RRID:AB_2756375	IHC (1:100)
Antibody	Anti-pSTAT1	Cell Signaling Technology	Cat# 9167 RRID:AB_561284	WB (1:100)
Antibody	Mouse monoclonal anti-IFNAR2 (clone MMHAR-2)	Thermo Fisher Scientific	Cat# 213851 RRID:AB_223508	Blocking (20 µg/mL) FACS (1 µg/mL)
Antibody	Mouse IgG2a isotype control	Thermo Fisher Scientific	Cat# 02-6200 RRID:AB_2532943	Blocking (20 µg/mL) IF (0.6 µg/mL)
Antibody	Polyclonal F(ab)2 goat anti‑rabbit IgG, AlexaFluor 555	Thermo Fisher Scientific	Cat# A-21430 RRID:AB_2535851	IF (2 µg/mL)
Antibody	Polyclonal F(ab)2 goat anti‑mouse IgG, AlexaFluor 488	Thermo Fisher Scientific	Cat# A-10684 RRID:AB_2534064	IF (2 µg/mL)
Antibody	Plyclonal F(ab)2 goat anti‑mouse IgG, AlexaFluor 555	Cell Signaling Technology	Cat# 4409 RRID:AB_1904022	IF (2 µg/mL)
Antibody	Polyclonal goat anti‑rabbit IgG, HRP	Thermo Fisher Scientific	Cat# 32460 RRID:AB_1185567	WB (1:10000)
Antibody	Polyclonal goat anti‑mouse IgG, HRP	Thermo Fisher Scientific	Cat# 31430 RRID:AB_228307	WB (1:10000)
Cytokine (recombinant, human)	M-CSF	Peprotech	Cat# 300‑25	(20 ng/mL)
Cytokine (recombinant, human)	IFNb	Peprotech	Cat# 300-02BC	10 and 100 U/mL
Cytokine (recombinant, human)	IL-10	Peprotech	Cat# 200-10	10 ng/mL
Monocyte isolation	Mouse anti‑human CD14 microbeads	Miltenyi Biotec	Cat# 130‑050‑201	
Monocyte isolation	LS magnetic columns	Miltenyi Biotec	Cat# 130‑042‑401	
Western blot	Amersham ECL Prisme Western Blotting Detection Reagent	GE Healthcare	Cat# RPN2232	
Western blot	SuperSignal WestPico Chemiluminescent Substrate	Thermo Scientific	Cat# 34080	
ELISA	IL‑10 ELISA set	BD Bioscience	Cat# 555157	
Cell culture	Trypsin EDTA 0.05%	Thermo Fisher Scientific	Cat# 25200072	
Cell culture	Accutase	Sigma-Aldrich	Cat# A-6964	
Probe	Phalloidin AlexaFluor 488	Thermo Fisher Scientific	Cat# A12379	(33 mM)
Probe	Phalloidin Alexa Fluor 647	Thermo Fisher Scientific	Cat# A22287	(33 mM)
Probe	DAPI	Sigma Aldrich	Cat# D9542	(500 ng/mL)
Probe	CellTracker Green CMFDA Dye	Thermo Fisher Scientific	Cat# C7025	(500 ng/mL)
Probe	MitoTracker Deep Red FM	Invitrogen	Cat# M22426	(500 ng/mL)
IF	Fluorescence Mounting Medium	Agilent Technologies	Cat# S302380‑2	
IF	Antibody diluent, Background reducing	DAKO, Agilent Technologies	Cat# S302283-2	
Software	ImageJ	ImageJ	http://www.imagej.nih.gov/ij	
Software	Prism (v8.0.0)	GraphPad	http://www.graphpad.com	
Software	Photoshop CS3	Adobe	http://www.adobe.com	
Software	Adobe Illustrator CS5	Adobe	https://www.adobe.com/fr/products/illustrator.html	
Software	Huygens Professional Version 16.10	Scientific Volume Imaging	https://svi.nl/HuygensProfessional	
Software	FACS DIVA	BD Bioscience	http://www.bdbiosciences.com/	
Software	FlowJo_v10	FlowJo	https://www.flowjo.com/	
Software	FCS Express V3	DeNovo Software	http://www.denovosoftware.com	
Software	Image Lab	Bio‑Rad Laboratories	http://www.bio‑rad.com	
Software	Pannoramic Viewer	3DHISTECH	https://www.3dhistech.com/pannoramic_viewer	

### Human subjects

Monocytes from healthy subjects were provided by Etablissement Français du Sang (EFS), Toulouse, France, under contract 21/PLER/TOU/IPBS01/20130042. According to articles L12434 and R124361 of the French Public Health Code, the contract was approved by the French Ministry of Science and Technology (agreement number AC 2009921). Written informed consents were obtained from the donors before sample collection.

### Non-Human primate (NHP) samples

All animal procedures were approved by the Institutional Animal Care and Use Committee of Tulane University, New Orleans, LA and were performed at the Tulane TNPRC, and under approval from IACUC (project numbers P3733, P3794, P3373 and P3628). They were performed in strict accordance with NIH guidelines. The twenty adult rhesus macaques used in this study (Supplementary file 1-Table S1 and S2) were bred and housed at the Tulane National Primate Research Center (TNPRC). All macaques were infected as previously described (Foreman et al., 2016; Mehra et al., 2011; Souriant et al., 2019). Briefly, aerosol infection was performed on macaques using a low dose (25 CFU implanted) of Mtb CDC1551. Nine weeks later, a subgroup of the animals was additionally intravenously injected with 300 TCID50 of SIVmac239 in 1 mL saline, while controls received an equal volume of saline solution. Euthanasia criteria were presentation of four or more of the following conditions: (i) body temperatures consistently greater than 2 °F above pre-infection values for three or more weeks in a row; (ii) 15% or more loss in body weight; (iii) serum CRP values higher than 10 mg/mL for three or more consecutive weeks, CRP being a marker for systemic inflammation that exhibits a high degree of correlation with active TB in macaques (Kaushal et al., 2012; Mehra et al., 2011); (iv) CXR values higher than two on a scale of 0–4; (v) respiratory discomfort leading to vocalization; (vi) significant or complete loss of appetite; and (vii) detectable bacilli in BAL samples.

### Bacteria

Mtb H37Rv strain was grown in suspension in Middlebrook 7H9 medium (Difco) supplemented with 10% albumin-dextrose-catalase (ADC, Difco) and 0.05% Twen-80 (Sigma-Aldrich) (Lastrucci et al., 2015). For infection, growing Mtb was centrifuged (3000 rpm) at exponential phase stage and resuspended in PBS (MgCl_2_, CaCl_2_ free, Gibco). Twenty passages through a 26 G needle were done for dissociation of bacterial aggregates. Bacterial suspension concentration was then determined by measuring OD_600_, and then resuspended in RPMI-1640 containing 10% FBS for infection.

### Viruses

Virus stocks were generated by transient transfection of 293 T cells with proviral plasmids coding for HIV-1 ADA and HIV-1 NLAD8-VSVG isolates, kindly provided by Serge Benichou (Institut Cochin, Paris, France), as previously described (Vérollet et al., 2015). Supernatant were harvested 2 days post-transfection and HIV-1 p24 antigen concentration was assessed by a homemade enzyme-linked immunosorbent assay (ELISA). HIV-1 infectious units were quantified, as reported (Souriant et al., 2019) using TZM-bl cells (NIH AIDS Reagent Program, Division of AIDS, NIAID, NIH from Dr. John C. Kappes, Dr. Xiaoyun Wu and Tranzyme Inc).

HIV-VLP stock (GFP VLP) was generated by transfecting the molecular clone pGag-eGFP obtained from the NIH AIDS Research and Reference Reagent Program. HEK-293 T cells were transfected with calcium phosphate (CalPhos, Clontech) in T75 flasks using 30 μg of plasmid DNA. Supernatants containing VLP were filtered (Millex HV, 0.45 μm; Millipore) and frozen at −80°C until use. The p24 Gag content of the VLP was determined by an ELISA (Perkin-Elmer).

### Preparation of human monocytes and monocyte-derived macrophages

Human monocytes were isolated from healthy subject (HS) buffy coat (from EFS) and differentiated towards macrophages as described (Souriant et al., 2019). Briefly, peripheral blood mononuclear cells (PBMC) were recovered by gradient centrifugation on Ficoll-Paque Plus (GE Healthcare). CD14^+^ monocytes were then isolated by positive selection magnetic sorting, using human CD14 Microbeads and LS columns (Miltenyi Biotec). Cells were then plated at 1.6 × 10^6^ cells per 6-well and allowed to differentiate for 5–7 days in RPMI-1640 medium (GIBCO), 10% Fetal Bovine Serum (FBS, Sigma-Aldrich) and human M-CSF (20 ng/mL) Peprotech) before infection with Mtb H37Rv for conditioned-media preparation. The cell medium was renewed every 3 or 4 days.

### Preparation of conditioned media

Conditioned-media from Mtb-infected macrophages (cmMTB) has been reported previously (Lastrucci et al., 2015; Souriant et al., 2019). Succinctly, hMDM were infected with Mtb H37Rv at a MOI of 3. After 18 hr of infection at 37°C, culture supernatants were collected, sterilized by double filtration (0.2 µm pores) and aliquots were stored at −80°C. We then tested the capacity of individual cmMTB to differentiate freshly isolated CD14^+^ monocytes towards the M(IL-10) cell-surface marker phenotype, as assessed by FACS analyses. Those supernatants yielding a positive readout were then pooled together (5–10 donors) to minimize the inter-variability obtained between donors. Control media (cmCTR) was obtained from uninfected macrophage supernatant. When specified, IL-10 was eliminated from cmMTB by antibody depletion as described previously (Lastrucci et al., 2015; Souriant et al., 2019). The depletion was verified by ELISA (BD-Bioscience), according to manufacturer’s protocol.

### Conditioning of monocytes with the secretome of Mtb-infected macrophages or cytokines

Human CD14^+^ sorted monocytes from HS buffy coat were allowed to adhere in the absence of serum (0.4 × 10^6^ cells / 24-well in 500 µL) on glass coverslips, and then cultured for 3 days with 40% dilution (vol/vol) of cmCTR or cmMTB supplemented with 20% FBS and M-CSF (20 ng/mL, Peprotech). Blocking IFNAR receptor was performed by pre-incubation with mouse anti-IFNAR antibody (20 µg/mL, Thermo Fischer Scientific) in a 200 μL for 30 min prior to conditioning. After 3 days, cells were washed and collected for phenotyping.

When specified, monocytes were also conditioned in presence of 20 ng/mL M-CSF and 10 ng/mL recombinant human IL-10 (PeproTech) or 10 U/mL of IFNβ (Peprotech). Cell-surface expression of Siglec-1 was measured by flow cytometry using standard procedures detailed hereafter.

### RNA extraction and transcriptomic analysis

Cells conditioned with cmCTR and cmMTB supernatants (approximately 1.5 million cells) were treated with TRIzol Reagent (Invitrogen) and stored at −80°C. Total RNA was extracted from the TRIzol samples using the RNeasy mini kit (Qiagen). RNA amount and purity (absorbance at 260/280 nm) was measured with the Nanodrop ND-1000 apparatus (Thermo Scientific). According to the manufacturer's protocol, complementary DNA was then reverse transcribed from 1 µg total RNA with Moloney murine leukemia virus reverse transcriptase (Invitrogen), using random hexamer oligonucleotides for priming. The microarray analysis was performed using the Agilent Human GE 4 × 44 v2 (single color), as previously described (Lugo-Villarino et al., 2018). Briefly, we performed hybridization with 2 μg Cy3-cDNA and the hybridization kit (Roche NimbleGen). The samples were then incubated for 5 min at 65°C, and 5 min at 42°C before loading for 17 hr at 42°C, according to manufacturer's protocol. After washing, the microarrays were scanned with MS200 microarray scanner (Roche NimbleGen), and using Feature Extraction software, the Agilent raw files were extracted and then processed through Bioconductor (version 3.1) in the R statistical environment (version 3.6.0). Using the limma package, raw expression values were background corrected in a ‘normexp’ fashion and then quantile normalized and log_2_ transformed (Ritchie et al., 2015). Density plots, boxplots, principal component analyses, and hierarchical clustering assessed the quality of the hybridizations. Differentially expressed genes between macrophages exposed to cmCTR or cmMTB supernatants were extracted based on the p-value corrected using the Benjamini-Hochberg procedure. The log_2_ normalized expression values were used to perform Gene Set Enrichment Analyses (GSEA). The GSEA method allows to statistically test whether a set of genes of interest (referred to as a geneset) is distributed randomly or not in the list of genes that were pre-ranked according to their differential expression ratio between macrophages exposed to cmCTR or cmMTB supernatants. The output of GSEA is a GeneSet enrichment plot. The vertical black lines represent the projection onto the ranked GeneList of the individual genes of the GeneSet. The top curve in green corresponds to the calculation of the enrichment score (ES). The more the ES curve is shifted to the upper left of the graph, the more the GeneSet is enriched in the red cell population. Conversely, the more the ES curve is shifted to the lower right of the graph, the more the GeneSet is enriched in the blue cell population.

### siRNA silencing

Targeted gene silencing in monocytes was performed using reverse transfection protocol as previously described (Troegeler et al., 2014). Shortly, human primary monocytes were transfected with 200 nM of ON-TARGETplus SMARTpool siRNA targeting Siglec-1 (Horizon Discovery) or non-targeting siRNA (control) using HiPerfect transfection system (Qiagen). After a four-hr post-transfection, cells were allowed to rest for 24 hr in RPMI-1640 medium, 10% FBS, 20 ng/mL of M-CSF, before addition of cmMTB media (40% vol/vol). After three additional days of conditioning, cells were infected with HIV-1 ADA or HIV-1- NLAD8-VSV-G strain, and kept in culture for 10 more days or 48 hrs, respectively. Validation of gene silencing was done after three days post-transfection, and this protocol led to the efficient depletion of Siglec-1 ranging between 50 to95%, as measured by flow cytometry.

### HIV-1 infection

For HIV-1 infection, at day 3 of differentiation, 0.4 × 10^6^ human monocytes-derived macrophages (hMDM) were infected with HIV-1 ADA strain (or as indicated otherwise) at MOI 0.1. HIV-1 infection, and replication were assessed at 10-day post-infection by measuring p24-positive cells by immunostaining and the level of p24 released in culture media by ELISA. For the infection and TNT quantification at day six post-infection, the same protocol was used. For HIV-1 transfer, higher MOI of HIV-1 VSVG pseudotyped NLAD8 virus was used, as described below (see section *HIV-1 and cell-to-cell transfer*) and in Souriant et al. (2019).

### Uptake of Virus-Like particles

Uptake experiment were performed as previously described (Izquierdo-Useros et al., 2012a; Izquierdo-Useros et al., 2014; Pino et al., 2015) using p24^Gag^ HIV-1_Gag−eGFP_ VLP (GFP VLP). Briefly, monocytes transfected (or not) with control siRNA, or with siRNA directed against Siglec-1, and differentiated for 3 days in cmCTR or cmMTB, were washed once with PBS prior to addition of 2 ng/mL of GFP VLP. Binding was performed during 3.5 hr at 37°C in a 5% CO_2_ incubator. Cells were then detached with cell dissociation buffer (Gibco) and prepared for flow cytometry analysis on a BD LSRFortessa (TRI-Genotoul platerform). Same experiment was also performed blocking monocyte-derived macrophages at RT for 15 min with 10 μg/mL of mAb α-Siglec-1 7–239 (Abcam), IgG1 isotype control (BD Biosciences) or leaving cells untreated before VLP addition.

### Flow cytometry and Siglec-1 quantitation

Staining of conditioned macrophages was performed as previously described (Souriant et al., 2019). Adherent cells were harvested after 5 min incubation in trypsin 0.05% EDTA (Gibco) and washes with PBS (Gibco). After 10 min centrifugation at 320 g, pellets were resuspended in cold staining buffer (PBS, 2 mM EDTA, 0.5% FBS) with fluorophore-conjugated antibodies (See Key ressources Table) and, in parallel, with the corresponding isotype control antibody using a general concentration of 1 µg/mL. After staining, cells were washed with cold staining buffer, centrifuged for 2 min at 320 g at 4°C, and analyzed by flow cytometry using BD LSRFortessa flow cytometer (BD Biosciences, TRI Genotoul plateform) and the associated BD FACSDiva software. Data were then analyzed using the FlowJo_V10 software (FlowJo, LLC). Gating on macrophage population was set according to its Forward Scatter (FSC) and Size Scatter (SSC) properties before doublet exclusion and analysis of the median fluorescence intensity (MFI) for each staining.

To determine Siglec-1 expression, we applied a quantitative FACS assay. Briefly, cmCTR- and cmMTB-treated macrophages were detached using Accutase solution (Gibco) for 10 min at 37°C, washed, blocked with 1 mg/mL human IgG (Privigen, Behring CSL), and stained with mAb 7–239 α-Siglec-1-PE or matched isotype-PE control (Biolegend) at 4°C for 30 min. The mean number of Siglec-1 mAb binding sites per cell was obtained with a Quantibrite kit (Becton Dickinson) as previously described (Izquierdo-Useros et al., 2012b). Samples were analyzed with FACSCalibur using CellQuest software to evaluate collected data.

### Immunofluorescence microscopy

Cells were fixed with PFA 3.7%, Sucrose 30 mM in PBS. After washing with PBS, cells were saturated with blocking buffer (PBS-BSA 1%). Depending on the experiments, cells were permeabilized as previously described (Souriant et al., 2019) with Triton X-100 0.3% for 10 min (or not), and then stained for 2 hrs with the primary antibody: anti-Siglec-1 (10 µg/mL, Novus Biologicals). Cells were then incubated with appropriate secondary antibodies for 1 hr: Alexa Fluor 488 or 555 or 647 Goat anti-Mouse IgG (2 µg/mL, Cell Signaling Technology). Cells were then permeabilized, washed in PBS before saturation with 0.6 µg/mL mouse IgG2 diluted in Dako Antibody Reducing Background buffer (Dako) for 30 min. Intracellular proteins were then stained with anti-Gag KC57 RD1 antibody (1/100, Beckman Coulter) and/or anti-α-tubulin (5 µg/mL, Abcam) for 2 hrs. Cells were washed and finally incubated with Alexa Fluor 488, 555 or 647 Goat anti-Mouse, or Goat anti-Rabbit IgG secondary antibodies (2 µg/mL, Cell Signaling Technology), Alexa Fluor 488 or 555 Phalloidin (33 mM, Thermo Fisher Scientific), Wheat Germ Agglutinin (CF350 WGA, Thermofischer) and DAPI (500 ng/mL, Sigma Aldrich) in blocking buffer for 1 hr. Coverslips were mounted on a glass slide using Fluorescence Mounting Medium (Dako) and visualized with a spinning disk (Olympus), a Zeiss confocal LSM880 with Airyscan or a FV1000 confocal microscope (Olympus).

TNT were identified by WGA or phalloidin and tubulin staining, and counted on at least 1000 cells per condition and per donor.

As HIV-1 infection induces macrophages fusion into MGC (Vérollet et al., 2010), the number of infected cells largely underestimates the rate of infection. Thus, to better reflect the rate of infection, we quantified the percentage of MGC. Using semi-automatic quantification with homemade Image J macros, allowing the study of more than 5,000 cells per condition in at least five independent donors, we assessed these parameters.

### HIV-1 and cell-to-cell transfer

Freshly isolated CD14^+^ monocytes from HS transfected with siRNA against Siglec-1 (or siRNA control) were allowed to adhere in the absence of serum (2 × 10^6^ cells/6-well in 1.5 mL). After 4 hr of culture, RPMI-1640 supplemented with 20 ng/mL M-CSF and 20% FBS were added to the cells (vol/vol). After 24 hr, cells were conditioned with cmMTB media. At day 4, 120 ng p24 of a HIV-1 NLAD8 strain pseudotyped with a VSVG envelope was used to infect half of the cells, kept in culture for two more days. At day 6, before co-culture, uninfected cells were stained with CellTracker Green CMFDA Dye (Thermo Fisher Scientific). For mitochondria transfer, half of the macrophages were pre-stained with Green CellTracker, and the other half, uninfected, was stained with mitoTracker Deep-Red prior to co-culture. Briefly, cells were washed with PBS Mg^2+^/Ca^2+^ and stained for 30 min with 500 ng/mL CellTracker or mitoTracker, before washing with RPMI-1640 10% FBS. HIV-1^+^(or mitoTracker^+^) and CellTracker^+^ cells were then detached using accutase (Sigma) and co-cultured at a 1:1 ratio on glass coverslips in 24-well.

### Histological analyses

Paraffin embedded tissue samples were sectioned and stained with hematoxylin and eosin for histomorphological analysis. Different antigen unmasking methods where used on tissue slides for immunohistochemical staining, which was performed using anti-CD163 (Leica/Novocastra), anti-Siglec-1 (Novus Biologicals) and anti-pSTAT1 (Cell Signaling Technology). Sections were then incubated with biotin-conjugated polyclonal anti-mouse or anti-rabbit immunoglobulin antibodies, followed by the streptavidin-biotin-peroxidase complex (ABC) method (Vector Laboratories). Finally, sections were counter-stained with hematoxylin. Slides were scanned with the Panoramic 250 Flash II (3DHISTECH). Virtual slides were automatically quantified for macrophage distribution as previously described (Souriant et al., 2019). Immunofluorescence staining of the sections was performed as described above, and followed by anti-mouse IgG isotype specific or anti-rabbit IgG antibodies labelled with Alexa488 and Alexa555 (Molecular Probes). Samples were mounted with Prolong Antifade reagent (Molecular Probes) and examined using a 60x/1.40N.A. objective of an Olympus spinning disk microscope.

### Quantification and statistical analysis

Information on the statistical tests used and the exact values of n (donors) can be found in the Figure Legends. All statistical analyses were performed using GraphPad Prism 8.0.0 (GraphPad Software Inc). Two-tailed paired or unpaired t-test was applied on data sets with a normal distribution (determined using Kolmogorov-Smirnov test), whereas two-tailed Mann-Whitney (unpaired test) or Wilcoxon matched-paired signed rank tests were used otherwise. p<0.05 was considered as the level of statistical significance (*p≤0.05; **p≤0.005; ***p≤0.0005; ****p≤0.0001).

## Data Availability

The raw data for the transcriptome analysis in this manuscript was made available through the public by a deposit to GEO under the accession code GSE139511. The following dataset was generated: MahnTPUGeanncarloL-V2020Tuberculosis-associated IFN-I induces Siglec-1 on microtubule-containing tunneling nanotubes and favors HIV-1 spread in macrophagesNCBI Gene Expression OmnibusGSE13951110.7554/eLife.52535PMC717396332223897

## Additional files

Supplementary file 1. Clinical data of NHPs (Table S1) and histopathological scoring of lung lesions in NHPs (Table S2).

Transparent reporting form

## References

[bib1] Akiyama H, Ramirez NG, Gudheti MV, Gummuluru S (2015). CD169-mediated trafficking of HIV to plasma membrane invaginations in dendritic cells attenuates efficacy of anti-gp120 broadly neutralizing antibodies. PLOS Pathogens.

[bib2] Akiyama H, Ramirez N-GP, Gibson G, Kline C, Watkins S, Ambrose Z, Gummuluru S (2017). Interferon-Inducible CD169/Siglec1 attenuates Anti-HIV-1 effects of alpha interferon. Journal of Virology.

[bib3] Bell LCK, Noursadeghi M (2018). Pathogenesis of HIV-1 and Mycobacterium tuberculosis co-infection. Nature Reviews Microbiology.

[bib4] Berry MP, Graham CM, McNab FW, Xu Z, Bloch SA, Oni T, Wilkinson KA, Banchereau R, Skinner J, Wilkinson RJ, Quinn C, Blankenship D, Dhawan R, Cush JJ, Mejias A, Ramilo O, Kon OM, Pascual V, Banchereau J, Chaussabel D, O'Garra A (2010). An interferon-inducible neutrophil-driven blood transcriptional signature in human tuberculosis. Nature.

[bib5] Bracq L, Xie M, Benichou S, Bouchet J (2018). Mechanisms for Cell-to-Cell transmission of HIV-1. Frontiers in Immunology.

[bib6] Cai Y, Sugimoto C, Liu DX, Midkiff CC, Alvarez X, Lackner AA, Kim WK, Didier ES, Kuroda MJ (2015). Increased monocyte turnover is associated with interstitial macrophage accumulation and pulmonary tissue damage in SIV-infected rhesus macaques. Journal of Leukocyte Biology.

[bib7] Cribbs SK, Lennox J, Caliendo AM, Brown LA, Guidot DM (2015). Healthy HIV-1-infected individuals on highly active antiretroviral therapy harbor HIV-1 in their alveolar macrophages. AIDS Research and Human Retroviruses.

[bib8] Crocker PR, Paulson JC, Varki A (2007). Siglecs and their roles in the immune system. Nature Reviews Immunology.

[bib9] Deffur A, Mulder NJ, Wilkinson RJ (2013). Co-infection with *Mycobacterium tuberculosis* and human immunodeficiency virus: an overview and motivation for systems approaches. Pathogens and Disease.

[bib10] Diedrich CR, Flynn JL (2011). HIV-1/*mycobacterium tuberculosis* coinfection immunology: how does HIV-1 exacerbate tuberculosis?. Infection and Immunity.

[bib11] Dupont M, Souriant S, Lugo-Villarino G, Maridonneau-Parini I, Vérollet C (2018). Tunneling nanotubes: intimate communication between myeloid cells. Frontiers in Immunology.

[bib12] Esmail H, Riou C, Bruyn ED, Lai RP, Harley YXR, Meintjes G, Wilkinson KA, Wilkinson RJ (2018). The immune response to *Mycobacterium tuberculosis* in HIV-1-Coinfected Persons. Annual Review of Immunology.

[bib13] Eugenin EA, Gaskill PJ, Berman JW (2009). Tunneling nanotubes (TNT) are induced by HIV-infection of macrophages: a potential mechanism for intercellular HIV trafficking. Cellular Immunology.

[bib14] Foreman TW, Mehra S, LoBato DN, Malek A, Alvarez X, Golden NA, Bucşan AN, Didier PJ, Doyle-Meyers LA, Russell-Lodrigue KE, Roy CJ, Blanchard J, Kuroda MJ, Lackner AA, Chan J, Khader SA, Jacobs WR, Kaushal D (2016). CD4 ^+^ T-cell–independent mechanisms suppress reactivation of latent tuberculosis in a macaque model of HIV coinfection. PNAS.

[bib15] Ganor Y, Real F, Sennepin A, Dutertre CA, Prevedel L, Xu L, Tudor D, Charmeteau B, Couedel-Courteille A, Marion S, Zenak AR, Jourdain JP, Zhou Z, Schmitt A, Capron C, Eugenin EA, Cheynier R, Revol M, Cristofari S, Hosmalin A, Bomsel M (2019). HIV-1 reservoirs in urethral macrophages of patients under suppressive antiretroviral therapy. Nature Microbiology.

[bib16] Gummuluru S, Pina Ramirez NG, Akiyama H (2014). CD169-dependent cell-associated HIV-1 transmission: a driver of virus dissemination. Journal of Infectious Diseases.

[bib17] Halász H, Ghadaksaz AR, Madarász T, Huber K, Harami G, Tóth EA, Osteikoetxea-Molnár A, Kovács M, Balogi Z, Nyitrai M, Matkó J, Szabó-Meleg E (2018). Live cell superresolution-structured illumination microscopy imaging analysis of the intercellular transport of microvesicles and costimulatory proteins via nanotubes between immune cells. Methods and Applications in Fluorescence.

[bib18] Hartnell A, Steel J, Turley H, Jones M, Jackson DG, Crocker PR (2001). Characterization of human sialoadhesin, a sialic acid binding receptor expressed by resident and inflammatory macrophage populations. Blood.

[bib19] Hashimoto M, Bhuyan F, Hiyoshi M, Noyori O, Nasser H, Miyazaki M, Saito T, Kondoh Y, Osada H, Kimura S, Hase K, Ohno H, Suzu S (2016). Potential role of the formation of tunneling nanotubes in HIV-1 spread in macrophages. The Journal of Immunology.

[bib20] Hekmatshoar Y, Nakhle J, Galloni M, Vignais ML (2018). The role of metabolism and tunneling nanotube-mediated intercellular mitochondria exchange in Cancer drug resistance. Biochemical Journal.

[bib21] Honeycutt JB, Wahl A, Baker C, Spagnuolo RA, Foster J, Zakharova O, Wietgrefe S, Caro-Vegas C, Madden V, Sharpe G, Haase AT, Eron JJ, Garcia JV (2016). Macrophages sustain HIV replication in vivo independently of T cells. Journal of Clinical Investigation.

[bib22] Honeycutt JB, Thayer WO, Baker CE, Ribeiro RM, Lada SM, Cao Y, Cleary RA, Hudgens MG, Richman DD, Garcia JV (2017). HIV persistence in tissue macrophages of humanized myeloid-only mice during antiretroviral therapy. Nature Medicine.

[bib23] Ivashkiv LB, Donlin LT (2014). Regulation of type I interferon responses. Nature Reviews Immunology.

[bib24] Izquierdo-Useros N, Lorizate M, Contreras FX, Rodriguez-Plata MT, Glass B, Erkizia I, Prado JG, Casas J, Fabriàs G, Kräusslich HG, Martinez-Picado J (2012a). Sialyllactose in viral membrane gangliosides is a novel molecular recognition pattern for mature dendritic cell capture of HIV-1. PLOS Biology.

[bib25] Izquierdo-Useros N, Lorizate M, Puertas MC, Rodriguez-Plata MT, Zangger N, Erikson E, Pino M, Erkizia I, Glass B, Clotet B, Keppler OT, Telenti A, Kräusslich HG, Martinez-Picado J (2012b). Siglec-1 is a novel dendritic cell receptor that mediates HIV-1 trans-infection through recognition of viral membrane gangliosides. PLOS Biology.

[bib26] Izquierdo-Useros N, Lorizate M, McLaren PJ, Telenti A, Kräusslich HG, Martinez-Picado J (2014). HIV-1 capture and transmission by dendritic cells: the role of viral glycolipids and the cellular receptor Siglec-1. PLOS Pathogens.

[bib27] Jambo KC, Banda DH, Kankwatira AM, Sukumar N, Allain TJ, Heyderman RS, Russell DG, Mwandumba HC (2014). Small alveolar macrophages are infected preferentially by HIV and exhibit impaired phagocytic function. Mucosal Immunology.

[bib28] Jaroenpool J, Rogers KA, Pattanapanyasat K, Villinger F, Onlamoon N, Crocker PR, Ansari AA (2007). Differences in the constitutive and SIV infection induced expression of siglecs by hematopoietic cells from non-human primates. Cellular Immunology.

[bib29] Kabaso D, Lokar M, Kralj-Iglič V, Veranič P, Iglič A (2011). Temperature and cholera toxin B are factors that influence formation of membrane nanotubes in RT4 and T24 urothelial Cancer cell lines. International Journal of Nanomedicine.

[bib30] Kaushal D, Mehra S, Didier PJ, Lackner AA (2012). The non-human primate model of tuberculosis. Journal of Medical Primatology.

[bib31] Kuroda MJ, Sugimoto C, Cai Y, Merino KM, Mehra S, Araínga M, Roy CJ, Midkiff CC, Alvarez X, Didier ES, Kaushal D (2018). High turnover of tissue macrophages contributes to tuberculosis reactivation in simian immunodeficiency Virus-Infected rhesus macaques. The Journal of Infectious Diseases.

[bib32] Lastrucci C, Bénard A, Balboa L, Pingris K, Souriant S, Poincloux R, Al Saati T, Rasolofo V, González-Montaner P, Inwentarz S, Moraña EJ, Kondova I, Verreck FA, Sasiain MC, Neyrolles O, Maridonneau-Parini I, Lugo-Villarino G, Cougoule C (2015). Tuberculosis is associated with expansion of a motile, permissive and immunomodulatory CD16(+) monocyte population via the IL-10/STAT3 Axis. Cell Research.

[bib33] Lokar M, Kabaso D, Resnik N, Sepčić K, Kralj-Iglič V, Veranič P, Zorec R, Iglič A (2012). The role of cholesterol-sphingomyelin membrane nanodomains in the stability of intercellular membrane nanotubes. International Journal of Nanomedicine.

[bib34] Lugo-Villarino G, Troegeler A, Balboa L, Lastrucci C, Duval C, Mercier I, Bénard A, Capilla F, Al Saati T, Poincloux R, Kondova I, Verreck FAW, Cougoule C, Maridonneau-Parini I, Sasiain MDC, Neyrolles O (2018). The C-Type lectin receptor DC-SIGN has an Anti-Inflammatory role in human M(IL-4) Macrophages in response to *Mycobacterium tuberculosis*. Frontiers in Immunology.

[bib35] Martinez-Picado J, McLaren PJ, Erkizia I, Martin MP, Benet S, Rotger M, Dalmau J, Ouchi D, Wolinsky SM, Penugonda S, Günthard HF, Fellay J, Carrington M, Izquierdo-Useros N, Telenti A (2016). Identification of Siglec-1 null individuals infected with HIV-1. Nature Communications.

[bib36] Martinez-Picado J, McLaren PJ, Telenti A, Izquierdo-Useros N (2017). Retroviruses as myeloid cell riders: what natural human Siglec-1 "Knockouts" Tell Us About Pathogenesis. Frontiers in Immunology.

[bib37] Mathews S, Branch Woods A, Katano I, Makarov E, Thomas MB, Gendelman HE, Poluektova LY, Ito M, Gorantla S (2019). Human Interleukin-34 facilitates microglia-like cell differentiation and persistent HIV-1 infection in humanized mice. Molecular Neurodegeneration.

[bib38] Mattila JT (2019). Type 1 interferon expression and signaling occur in spatially-distinct regions in granulomas from Mycobacterium tuberculosis-infected cynomolgus macaques. The Journal of Immunology.

[bib39] McNab F, Mayer-Barber K, Sher A, Wack A, O'Garra A (2015). Type I interferons in infectious disease. Nature Reviews Immunology.

[bib40] Mehra S, Golden NA, Dutta NK, Midkiff CC, Alvarez X, Doyle LA, Asher M, Russell-Lodrigue K, Monjure C, Roy CJ, Blanchard JL, Didier PJ, Veazey RS, Lackner AA, Kaushal D (2011). Reactivation of latent tuberculosis in rhesus macaques by coinfection with simian immunodeficiency virus. Journal of Medical Primatology.

[bib41] Merino KM, Allers C, Didier ES, Kuroda MJ (2017). Role of monocyte/Macrophages during HIV/SIV infection in adult and pediatric acquired immune deficiency syndrome. Frontiers in Immunology.

[bib42] Moreira-Teixeira L, Mayer-Barber K, Sher A, O'Garra A (2018). Type I interferons in tuberculosis: foe and occasionally friend. Journal of Experimental Medicine.

[bib43] O'Garra A, Redford PS, McNab FW, Bloom CI, Wilkinson RJ, Berry MP (2013). The immune response in tuberculosis. Annual Review of Immunology.

[bib44] O'Neill ASG, van den Berg TK, Mullen GED (2013). Sialoadhesin - a macrophage-restricted marker of immunoregulation and inflammation. Immunology.

[bib45] Onfelt B, Nedvetzki S, Benninger RK, Purbhoo MA, Sowinski S, Hume AN, Seabra MC, Neil MA, French PM, Davis DM (2006). Structurally distinct membrane nanotubes between human macrophages support long-distance vesicular traffic or surfing of Bacteria. The Journal of Immunology.

[bib46] Osteikoetxea-Molnár A, Szabó-Meleg E, Tóth EA, Oszvald Á, Izsépi E, Kremlitzka M, Biri B, Nyitray L, Bozó T, Németh P, Kellermayer M, Nyitrai M, Matko J (2016). The growth determinants and transport properties of tunneling nanotube networks between B lymphocytes. Cellular and Molecular Life Sciences.

[bib47] Pino M, Erkizia I, Benet S, Erikson E, Fernández-Figueras MT, Guerrero D, Dalmau J, Ouchi D, Rausell A, Ciuffi A, Keppler OT, Telenti A, Kräusslich HG, Martinez-Picado J, Izquierdo-Useros N (2015). HIV-1 immune activation induces Siglec-1 expression and enhances viral trans-infection in blood and tissue myeloid cells. Retrovirology.

[bib48] Puryear WB, Yu X, Ramirez NP, Reinhard BM, Gummuluru S (2012). HIV-1 incorporation of host-cell-derived glycosphingolipid GM3 allows for capture by mature dendritic cells. PNAS.

[bib49] Puryear WB, Akiyama H, Geer SD, Ramirez NP, Yu X, Reinhard BM, Gummuluru S (2013). Interferon-inducible mechanism of dendritic cell-mediated HIV-1 dissemination is dependent on Siglec-1/CD169. PLOS Pathogens.

[bib50] Redondo-Morata L, Giannotti MI, Sanz F (2012). Influence of cholesterol on the phase transition of lipid bilayers: a Temperature-Controlled force spectroscopy study. Langmuir.

[bib51] Rempel H, Calosing C, Sun B, Pulliam L (2008). Sialoadhesin expressed on IFN-induced monocytes binds HIV-1 and enhances infectivity. PLOS ONE.

[bib52] Ritchie ME, Phipson B, Wu D, Hu Y, Law CW, Shi W, Smyth GK (2015). Limma powers differential expression analyses for RNA-sequencing and microarray studies. Nucleic Acids Research.

[bib53] Rodrigues V, Ruffin N, San-Roman M, Benaroch P (2017). Myeloid cell interaction with HIV: a complex relationship. Frontiers in Immunology.

[bib54] Rustom A, Saffrich R, Markovic I, Walther P, Gerdes HH (2004). Nanotubular highways for intercellular organelle transport. Science.

[bib55] Sattentau QJ, Stevenson M (2016). Macrophages and HIV-1: an unhealthy constellation. Cell Host & Microbe.

[bib56] Schneider WM, Chevillotte MD, Rice CM (2014). Interferon-stimulated genes: a complex web of host defenses. Annual Review of Immunology.

[bib57] Sewald X, Ladinsky MS, Uchil PD, Beloor J, Pi R, Herrmann C, Motamedi N, Murooka TT, Brehm MA, Greiner DL, Shultz LD, Mempel TR, Bjorkman PJ, Kumar P, Mothes W (2015). Retroviruses use CD169-mediated trans-infection of permissive lymphocytes to establish infection. Science.

[bib58] Souriant S, Balboa L, Dupont M, Pingris K, Kviatcovsky D, Cougoule C, Lastrucci C, Bah A, Gasser R, Poincloux R, Raynaud-Messina B, Al Saati T, Inwentarz S, Poggi S, Moraña EJ, González-Montaner P, Corti M, Lagane B, Vergne I, Allers C, Kaushal D, Kuroda MJ, Sasiain MDC, Neyrolles O, Maridonneau-Parini I, Lugo-Villarino G, Vérollet C (2019). Tuberculosis exacerbates HIV-1 infection through IL-10/STAT3-Dependent tunneling nanotube formation in macrophages. Cell Reports.

[bib59] Subramanian A, Tamayo P, Mootha VK, Mukherjee S, Ebert BL, Gillette MA, Paulovich A, Pomeroy SL, Golub TR, Lander ES, Mesirov JP (2005). Gene set enrichment analysis: a knowledge-based approach for interpreting genome-wide expression profiles. PNAS.

[bib60] Thayanithy V, Babatunde V, Dickson EL, Wong P, Oh S, Ke X, Barlas A, Fujisawa S, Romin Y, Moreira AL, Downey RJ, Steer CJ, Subramanian S, Manova-Todorova K, Moore MA, Lou E (2014). Tumor exosomes induce tunneling nanotubes in lipid raft-enriched regions of human mesothelioma cells. Experimental Cell Research.

[bib61] Torralba D, Baixauli F, Sánchez-Madrid F (2016). Mitochondria know no boundaries: mechanisms and functions of intercellular mitochondrial transfer. Frontiers in Cell and Developmental Biology.

[bib62] Tóth EA, Oszvald Ádám, Péter M, Balogh G, Osteikoetxea-Molnár A, Bozó T, Szabó-Meleg E, Nyitrai M, Derényi I, Kellermayer M, Yamaji T, Hanada K, Vígh L, Matkó J (2017). Nanotubes connecting B lymphocytes: high impact of differentiation-dependent lipid composition on their growth and mechanics. Biochimica Et Biophysica Acta (BBA) - Molecular and Cell Biology of Lipids.

[bib63] Troegeler A, Lastrucci C, Duval C, Tanne A, Cougoule C, Maridonneau-Parini I, Neyrolles O, Lugo-Villarino G (2014). An efficient siRNA-mediated gene silencing in primary human monocytes, dendritic cells and macrophages. Immunology and Cell Biology.

[bib64] VanderVen BC, Huang L, Rohde KH, Russell DG (2016). The minimal unit of infection: mycobacterium tuberculosis in the macrophage. Microbiology Spectrum.

[bib65] Vérollet C, Zhang YM, Le Cabec V, Mazzolini J, Charrière G, Labrousse A, Bouchet J, Medina I, Biessen E, Niedergang F, Bénichou S, Maridonneau-Parini I (2010). HIV-1 nef triggers macrophage fusion in a p61Hck- and protease-dependent manner. The Journal of Immunology.

[bib66] Vérollet C, Souriant S, Bonnaud E, Jolicoeur P, Raynaud-Messina B, Kinnaer C, Fourquaux I, Imle A, Benichou S, Fackler OT, Poincloux R, Maridonneau-Parini I (2015). HIV-1 reprograms the migration of macrophages. Blood.

[bib67] Yu YR, Hotten DF, Malakhau Y, Volker E, Ghio AJ, Noble PW, Kraft M, Hollingsworth JW, Gunn MD, Tighe RM (2016). Flow cytometric analysis of myeloid cells in human blood, Bronchoalveolar Lavage, and lung tissues. American Journal of Respiratory Cell and Molecular Biology.

[bib68] Ziegler-Heitbrock L, Lötzerich M, Schaefer A, Werner T, Frankenberger M, Benkhart E (2003). IFN-alpha induces the human IL-10 gene by recruiting both IFN regulatory factor 1 and Stat3. The Journal of Immunology.

[bib69] Zou Z, Chastain A, Moir S, Ford J, Trandem K, Martinelli E, Cicala C, Crocker P, Arthos J, Sun PD (2011). Siglecs facilitate HIV-1 infection of macrophages through adhesion with viral sialic acids. PLOS ONE.

