# Supplementary material for: Tuberculosis-associated IFN-I induces Siglec-1 on tunneling nanotubes and favors HIV-1 spread in macrophages
Source: eLife. 2020 Mar 30;9:e52535. doi: 10.7554/eLife.52535 (PMC7173963; doi:10.7554/eLife.52535)
Supplement: Supplementary file 1. [file elife-52535-supp1.docx]

**SUPPLEMENTARY TABLES**

**Supplementary File - Table S1. Clinical data of NHPs**.

| **Animals** | **Status** | **Sex** | **Age of death (years)** | **Days after infection*** | **Viral load**** |
| --- | --- | --- | --- | --- | --- |
| **EC61** | - | M | 8.99 | - | - |
| **GI53** | - | M | 5.02 | - | - |
| **IT02** | - | M | 4.69 | - | - |
| **BK48** | SIV | M | 12.06 | 322 | 8.58 x 10^6^ |
| **DD87** | SIV | F | 9.84 | 540 | 3.03 x 10^4^ |
| **DT18** | SIV | M | 8.85 | 764 | 4.40 x 10^4^ |
| **BA34** | SIV | M | 16.79 | 750 | 1.91 x 10^5^ |
| **DR28** | SIV | M | 8.84 | 120 | 1.48 x 10^7^ |
| **CA75** | LTB | M | 11.7 | 106 | - |
| **FE10** | LTB | M | 7 | 166 | - |
| **FJ05** | LTB | M | 6.81 | 181 | - |
| **CL10** | ATB | M | 13.84 | 51 | - |
| **CG58** | ATB | M | 7.71 | 75 | - |
| **GK87** | ATB | F | 7.76 | 38 | - |
| **ER44** | SIV/LTB | M | 8.61 | 167 | 1.93 x 10^5^ |
| **HB12** | SIV/LTB | M | 4.97 | 167 | 1.04 x 10^6^ |
| **ID01** | SIV/LTB | M | 3.58 | 153 | 2.17 x 10^6^ |
| **HP22** | SIV/ATB | M | 3.79 | 113 | 3.7 x 10^8^ |
| **HP41** | SIV/ATB | M | 3.78 | 111 | 6 x 10^5^ |
| **HT09** | SIV/ATB | M | 3.72 | 104 | 7.5 x 10^6^ |

* Infection 1: Mtb CDC1551 and infection 2: SIVmac239. For co-infected macaques, days after first Mtb infection. Abbreviations: LTB: latent TB, ATB: active TB, M: male, F: female.

**Supplementary File - Table S2. Histopathological scoring of lung lesions in NHPs**.

| **Animals** | **Status** | **Lung Disease severity** | **Granulomatous lesions** | | | | **Non- Granulomatous lesions** |
| --- | --- | --- | --- | --- | --- | --- | --- |
|  |  |  | **Granuloma Size** | **Type of granuloma** | **Distribution pattern** | **Cellular composition** |  |
| **EC61** | - | None | - | - | - | - | - Few & small perivascular lymphohistiocytic infiltrates, PMN+ |
| **GI53** | - | None | - | - | - | - | - Few & small perivascular lymphohistiocytic infiltrates, PMN+ |
| **IT02** | - | None | - | - | - | - | - Few & small perivascular lymphohistiocytic infiltrates  -PMN+  -peribronchial iBALT |
| **BK48** | SIV | Minimal | - | - | - | - | - small perivascular lymphohistiocytic infiltrates |
| **DD87** | SIV | Minimal | - | - | - | - | - small perivascular lymphohistiocytic infiltrates |
| **DT18** | SIV | Minimal | - | - | - | - | -polymorph infiltrates |
| **BA34** | SIV | Mild | - | - | - | - | - Focal interstitial pneumonia,  - interstitial lymphohistiocytic infiltrates,  - thickening of the alveolar wall,  - collagen deposit, - type-2 pneumocyte hyperplasia |
| **DR28** | SIV | Minimal | - | - | - | - | thickening of the alveolar wall closed to the pleura |
| **CA75** | LTB | Mild | small | Non  Necrotizing,  Poorly organized | multifocal | - Lymphocytic cuff  - Epithelioid & Foamy Mφ  - MGC, - Fibrosis [+] | Interstitial pneumopathy |
| **FE10** | LTB | Mild | large | Nonnecrotizing  Suppurative | focal solid coalescent | - Lymphocytic cuff  - Epithelioid & Foamy Mφ  - MGC | Thickening of the alveolar wall |
| **FJ05** | LTB | Mild | medium | caseous | multifocal | - Lymphocytic cuff  - peripheral fibrosis | Strong interstitial pneumopathy |
| **CL10** | ATB | Moderate | Small & medium | Caseous & solid | Multifocal coalescent | - Lymphocytic cuff  - Epithelioid Mφ, - MGC++ | - Strong interstitial pneumopathy  & haemorrhage |
| **CG58** | ATB | Mild | Small, medium & large | Necrotic & solid | Multifocal coalescent | - Lymphocytic cuff  - Epithelioid Mφ, - MGC++ | - |
| **GK87** | ATB | Moderate | Medium & large | Necrotic, caseous and supurative | Multifocal coalescent | - Lymphocytic cuff  - Epithelioid Mφ, MGC++, PMN++ | -transudat in the alveolar space,  - collagen deposits  -Interstitial pneumopathy |
| **ER44** | SIV/LTB | Mild | large | Necrotic & caseous | focal | 2 coalescent follicles,  Loss of the lymphocytic cuff,  Epithelioid Mφ- MGC++, | -Interstitial pneumopathy,  - haemorrhage & fibrosis |
| **HB12** | SIV/LTB | Severe | none | - | - | - | -Interstitial pneumopathy, iBALT  - haemorrhage & alveolitis,  - fibrosis, syncitia and PMN++ |
| **ID01** | SIV/LTB | Moderate | none | - | - | - | -Interstitial pneumopathy, iBALT  - haemorrhage & alveolitis, |
| **HP22** | SIV/ATB | Moderate | medium | Solid, necrotic, fibrotic & mineralized | multifocal | - Lymphocytic cuff  - Epithelioid Mφ, MGC++ | -Interstitial pneumopathy  - haemorrhage & syncitia |
| **HP41** | SIV/ATB | Severe | large | Necrotic | coalescent | - Lymphocytic cuff  - Epithelioid Mφ, MGC++ | -strong Interstitial pneumopathy,  - haemorrhage & alveolitis, fibrosis, PMN++ |
| **HT09** | SIV/ATB | Severe | large | Supurative | Multifocal, coalscent & invasive | Loss of the lymphocytic cuff,  Epithelioid Mφ, MGC++, PMN++  fibrosis | - Interstitial pneumopathy,  - haemorrhage |

Abbreviations: PMN: ploymorphonuclear leukocytes, iBALT: inducible Bronchus-associated lymphoid tissue, Mϕ: Macrophages, MGC: Multinucleated giant cells.
