# Supplementary material for: Tuberculosis-associated IFN-I induces Siglec-1 on tunneling nanotubes and favors HIV-1 spread in macrophages
Source: eLife. 2020 Mar 30;9:e52535. doi: 10.7554/eLife.52535 (PMC7173963; doi:10.7554/eLife.52535)
Supplement: Transparent reporting form [file elife-52535-transrepform.docx]

***eLife’s* transparent reporting form**

We encourage authors to provide detailed information *within their submission* to facilitate the interpretation and replication of experiments. Authors can upload supporting documentation to indicate the use of appropriate reporting guidelines for health-related research (see [EQUATOR Network](http://www.equator-network.org/%20)), life science research (see the [BioSharing Information Resource](https://biosharing.org/)), or the [ARRIVE guidelines](http://www.plosbiology.org/article/info:doi/10.1371/journal.pbio.1000412) for reporting work involving animal research. Where applicable, authors should refer to any relevant reporting standards documents in this form.

If you have any questions, please consult our Journal Policies and/or contact us: [editorial@elifesciences.org](mailto:editorial@elifesciences.org).

**Sample-size estimation**

- You should state whether an appropriate sample size was computed when the study was being designed
- You should state the statistical method of sample size computation and any required assumptions
- If no explicit power analysis was used, you should describe how you decided what sample (replicate) size (number) to use

Please outline where this information can be found within the submission (e.g., sections or figure legends), or explain why this information doesn’t apply to your submission:

For human data, the monocytes from healthy subjects were provided by Etablissement Français du Sang (EFS), Toulouse, France, under contract 21/PLER/TOU/IPBS01/20130042. According to articles L12434 and R124361 of the French Public Health Code, the contract was approved by the French Ministry of Science and Technology (agreement number AC 2009921). Written informed consents were obtained from the donors before sample collection. The number of independent experiments and donors was assessed based on our previous work: Souriant *et al.*, **Cell Reports**, 2019 (PMID: 30917314), and Lastrucci *et al*., **Cell Research**, 2015 (PMID: 26482950). When possible (e.g., Figure 1B, C), all donors tested during the various independent experiments involving the same readout were included in order to increase statistical power. Of note, in our experience, the minimum number of independent donors employed for each data point is N=5, but most included N=6-10.

For the non-human primate data, we obtained lung biopsies from our collaborators (Tulane National Primate Research Center) that were originally published in 2011 (Mehra *et al.*, **J Med Primatol**; PMID: 21781131). Twenty-one adult rhesus macaques were used in this study, where three animals were used per experimental group (seven groups). This minimal number of animals was determined and approved by the Institutional Animal Care and Use Committee of Tulane University, New Orleans (LA), and were performed in strict accordance with NIH guidelines.

This study does not include any other animal model such as rodents. In addition, no specific method or statistical tool were used for Sample-size estimation.

**Replicates**

- You should report how often each experiment was performed
- You should include a definition of biological versus technical replication
- The data obtained should be provided and sufficient information should be provided to indicate the number of independent biological and/or technical replicates
- If you encountered any outliers, you should describe how these were handled
- Criteria for exclusion/inclusion of data should be clearly stated
- High-throughput sequence data should be uploaded before submission, with a private link for reviewers provided (these are available from both GEO and ArrayExpress)

Please outline where this information can be found within the submission (e.g., sections or figure legends), or explain why this information doesn’t apply to your submission:

The transcriptome analysis was all included in the GEO Submission (GSE139511), which will be made publicly once this manuscript is accepted in this journal or elsewhere. Should the reviewers need to access these data files, we will be more than happy to provide tokens upon request.

All experiments of flow cytometry, RT-qPCR and infection quantitation (HIV binding, HIV replication) were performed independently at least three times, using two independent donors for comparison on a given day. At minimum, five donors in total were used to assess the statistical significance of our observations. This information can be found in the methods section and the figure legend at the end of each panel description.

Biological replication was defined as the repetition of the same experiment during different times using different blood donors. Technical replication was considered as the repetition of the same experiment on the same day, using two donors at the same time. In addition, internal replicates were performed by duplicating conditions for one given readout per donor.

Throughout the paper, we used (except for tunneling nanotubes (TNT) quantification) at least five independent donors (technical replicate) performed in at least three independent experiments (biological replicate).

Outliers were found in certain experiments and ruled out for specific substantiating reasons: i) cells that did not acquire the expected phenotype after conditioning, and ii) gene silencing was not efficient (below 50% efficiency, donors were excluded).

This information can be found in the material and methods section.

**Statistical reporting**

- Statistical analysis methods should be described and justified
- Raw data should be presented in figures whenever informative to do so (typically when N per group is less than 10)
- For each experiment, you should identify the statistical tests used, exact values of N, definitions of center, methods of multiple test correction, and dispersion and precision measures (e.g., mean, median, SD, SEM, confidence intervals; and, for the major substantive results, a measure of effect size (e.g., Pearson's r, Cohen's d)
- Report exact p-values wherever possible alongside the summary statistics and 95% confidence intervals. These should be reported for all key questions and not only when the p-value is less than 0.05.

Please outline where this information can be found within the submission (e.g., sections or figure legends), or explain why this information doesn’t apply to your submission:

Statistical analyses were performed as follow for all human data:

- D'Agostino & Pearson (A&P) or Kolmogorov-Smirnov (KS) normality test (depending on N size) on the groups being compared was performed.
- If both groups presented normal distribution, two-tailed paired t-test was applied;
- If either one group or none had a normal distribution, Wilcoxon matched-pairs

signed rank test was applied.

Groups were paired since we compared the same read out in cells originating from the same donor and treated under 2 different conditions. For TNT length and staining comparison, between 120 and 400 TNT were measured in at least 1,000 cells for 2 independent donors. After A&P or KS normality testing, non-normal distribution of the samples was analyzed with the non-paired Mann-Whitney test. The presence of Siglec-1 on the different categories of TNT was analyzed by a two-way ANOVA.

For NHP samples, statistical analysis was performed as follows:

- % of Siglec-1 or pSTAT1 positive alveolar macrophages and % of Siglec-1 positive cells in the lungs were assessed for A&P or KS normal distribution and failed. Consequently, an unpaired Mann-Whitney test was applied.
- Correlation curves were analyzed using Spearman test because data were non-parametric due to the small sample size (low number of animals per group).

All information on statistical test are available in the figure legends.

Raw data are presented in the majority of the graphs by a symbol, each one representing the value of the specific readout per donor, while the bar represents the mean value. The N is also given in the figure legend for each panel.

Exact p-values are reported in the annex file with all raw data.

(For large datasets, or papers with a very large number of statistical tests, you may upload a single table file with tests, Ns, etc., with reference to sections in the manuscript.)

**Group allocation**

- Indicate how samples were allocated into experimental groups (in the case of clinical studies, please specify allocation to treatment method); if randomization was used, please also state if restricted randomization was applied
- Indicate if masking was used during group allocation, data collection and/or data analysis

Please outline where this information can be found within the submission (e.g., sections or figure legends), or explain why this information doesn’t apply to your submission:

Not applicable. We did not perform any clinical trial. The data from the non-human primate groups was generated from histology analyses of lung biopsies obtained from our collaborators (Tulane National Primate Research Center), which belong originally to a study published in 2011 (Mehra et al., **J Med Primatol**; PMID: 21781131).

**Additional data files (“source data”)**

- We encourage you to upload relevant additional data files, such as numerical data that are represented as a graph in a figure, or as a summary table
- Where provided, these should be in the most useful format, and they can be uploaded as “Source data” files linked to a main figure or table
- Include model definition files including the full list of parameters used
- Include code used for data analysis (e.g., R, MatLab)
- Avoid stating that data files are “available upon request”

Please indicate the figures or tables for which source data files have been provided:

Figure 1A: GEO Submission (GSE139511)

Figure 1B-D to F-H: Excel file in annex

Figure 2A-D to F, H: Excel file in annex

Figure 3B, C, E: Excel file in annex

Figure 4B-E and G: Excel file in annex

Figure S4A-E, G: Excel file in annex
